# Phosphorylated STYK1 Promotes Gemcitabine Resistance via Autophagy‐Mediated Cancer Stemness in Pancreatic Cancer

**DOI:** 10.1002/advs.202521488

**Published:** 2026-10-11

**Authors:** Xueying Dong, Shi Li, Chensong Huang, Xuehong Qian, Jieyu Guo, Yahao Guo, Guifang Wu, Qi Zhang, Hongfei Tan, Yu Han, Rui Zhang, Hao Lyu, Shuai Xiao, Dong Guo, Chaojun Yan, Wenying Qin, Changchun Li, Xing‐Zhen Chen, Ying Zhao, Cefan Zhou, Jingfeng Tang

**Affiliations:** ^1^ National “111” Center for Cellular Regulation and Molecular Pharmaceutics Key Laboratory of Fermentation Engineering (Ministry of Education) Hubei University of Technology Wuhan China; ^2^ Hubei Key Laboratory of Resource Utilization and Quality Control of Characteristic Crops, College of Life Science and Technology Hubei Engineering University Xiaogan China; ^3^ Department of Pancreato‐Biliary Surgery The First Affiliated Hospital of Sun Yat‐Sen University Guangzhou China; ^4^ Institute of Health and Medicine Hefei Comprehensive National Science Center Hefei Anhui China; ^5^ Membrane Protein Disease Research Group Department of Physiology Faculty of Medicine and 8 Dentistry University of Alberta Edmonton Alberta Canada; ^6^ State Key Laboratory of Molecular Oncology Frontiers Science Center For Cancer Integrative Omics Peking University Cancer Hospital and Institute Peking University Health Science Center Beijing China

**Keywords:** AMPK, autophagy, cancer stemness, pancreatic gemcitabine resistance, STYK1

## Abstract

Pancreatic ductal adenocarcinoma (PDAC) is one of the most lethal malignancies, with gemcitabine resistance remaining a major clinical challenge. Although cancer stemness and autophagy have both been implicated in PDAC chemoresistance, the mechanism by which their crosstalk governs gemcitabine resistance remains poorly understood. Here, we identify serine/threonine/tyrosine kinase 1 (STYK1) as a critical mediator of gemcitabine resistance through autophagy‐ dependent maintenance of pancreatic cancer stemness. Mechanistically, gemcitabine activates AMP‐activated protein kinase (AMPK), which contributes to the enhancement of STYK1‐mediated autophagic flux, thereby promoting stemness‐associated chemoresistance. Furthermore, AMPK phosphorylates STYK1 at residues Ser91 and Ser304, thereby further potentiating STYK1‐mediated autophagy and cancer stemness. Importantly, we developed SAIDP5, a therapeutic peptide disrupting STYK1‐AMPK binding, which synergizes with gemcitabine to attenuate chemoresistance. This study reveals that gemcitabine‐induced AMPK activation amplifies STYK1‐driven autophagy and cancer stemness, thereby contributing to gemcitabine resistance, and highlights SAIDP5 as a promising peptide‐based strategy to improve gemcitabine efficacy in PDAC.

## Graphical abstract

Gemcitabine resistance remains a major challenge in pancreatic ductal adenocarcinoma. This study identifies STYK1 as a key mediator of chemoresistance through autophagy‐dependent maintenance of cancer stemness. Gemcitabine‐activated AMPK phosphorylates STYK1 at Ser91 and Ser304, enhancing autophagy and stemness. The therapeutic peptide SAIDP5 disrupts STYK1–AMPK binding and sensitizes PDAC cells to gemcitabine.

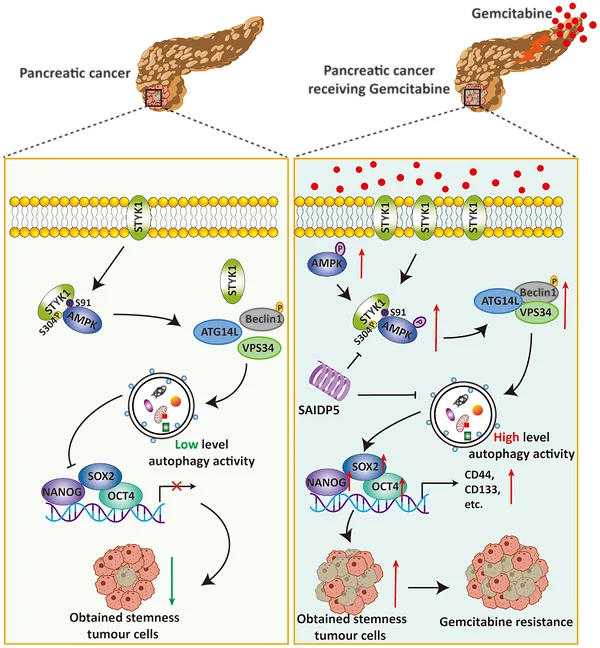

## 1 Introduction

Pancreatic ductal adenocarcinoma (PDAC), accounting for over 90% of all pancreatic malignancies, remains one of the most lethal human cancers, with a dismal 5‐year survival rate of approximately 12% [1, 2, 3]. This poor prognosis is attributable to its asymptomatic onset, a lack of reliable early diagnostic biomarkers, intrinsic and acquired therapeutic resistance, and limited effective treatment strategies. The unique tumor microenvironment (TME) and remarkable biological heterogeneity of PDAC present significant therapeutic challenges. Adjuvant combination chemotherapy following surgical resection plays a crucial role in the treatment of early‐stage pancreatic cancer. Gemcitabine serves as a first‐line chemotherapeutic agent, and its efficacy can be enhanced by combination with nab‐paclitaxel or tegafur‐based regimens [4]. Nevertheless, the development of chemoresistance remains the primary determinant of unfavorable clinical outcomes. Gemcitabine resistance arises through multiple mechanisms, including epithelial‐mesenchymal transition (EMT), cancer stemness, apoptotic evasion, metabolic remodeling, autophagy activation, immune‐suppressive reprogramming, and dysregulation of critical signaling pathways [5, 6, 7]. These molecular adaptations collectively compromise the efficacy of targeted therapeutic interventions, highlighting the imperative for novel therapeutic targets and agents capable of overcoming chemoresistance.

Cancer stemness, characterized by self‐renewal capacity, multilineage differentiation potential, and intrinsic therapeutic resistance, constitutes the principal pathogenic driver of PDAC progression and acquired chemoresistance [8, 9, 10]. In PDAC, elevated stemness is characterized by overexpression of surface markers such as CD44 and CD133, as well as enhanced aldehyde dehydrogenase 1 (ALDH1) activity [11], which collectively enable tumor cells to evade cytotoxic stress. This malignant phenotype is sustained through core transcriptional regulators (such as OCT4, NANOG, SOX2) [10] and evolutionarily conserved signaling pathways, including Wnt/β‐catenin, Hedgehog, JAK/STAT, PI3K/AKT, Notch, and Hippo signaling [9, 12, 13, 14, 15, 16, 17, 18]. Recent studies have further emphasized that crosstalk between Notch, Hedgehog, Wnt/β‐catenin, TGF‐β, and JAK/STAT signaling networks contributes to cancer stemness maintenance, metastatic potential, and therapeutic resistance [6]. Clinically, high stemness correlates with poor prognosis, metastatic dissemination, and treatment failure in PDAC [19, 20, 21, 22, 23], suggesting that therapeutic strategies targeting cancer stemness could yield significant clinical benefits.

Autophagy, an evolutionarily conserved lysosomal degradation pathway, is essential for clearing misfolded proteins and damaged organelles, thereby maintaining cellular homeostasis and supporting stress adaptation [24, 25, 26]. Autophagy initiation involves ATG‐dependent formation of double‐membraned autophagosomes that sequester cargo for lysosomal degradation [25, 27, 28]. Notably, autophagy exerts context‐dependent pro‐ and anti‐tumor effects during cancer progression and is intimately linked to tumorigenesis and chemotherapy response [29]. This dual role has recently been emphasized in PDAC, where autophagy can suppress early tumor initiation but also support tumor cell survival, metabolic adaptation, and resistance under therapeutic stress [30]. PDAC is characterized by elevated autophagic flux, and autophagy inhibition has been shown to suppress tumor growth through mechanisms including attenuation of reactive oxygen species (ROS), mitigation of DNA damage, and reprogramming of cellular metabolism [23, 31]. Consistently, genetic blockade of autophagy‐related components, including ULK1 and ATG7, has been reported to inhibit pancreatic cancer progression and enhance chemosensitivity in drug‐resistant pancreatic cancer cells [32, 33]. Importantly, cancer stemness is sustained by heightened autophagic activity, as evidenced by upregulation of ATG5 and Beclin1 [34, 35]. A recent review further supports that protective autophagy promotes cancer stemness, drug resistance, and maintenance of stemness‐associated markers, including in pancreatic cancer models [36]. Additionally, autophagy maintains metabolic plasticity by recycling damaged organelles to provide energy and nutrients, while concurrently regulating core stemness pathways such as Wnt/β‐catenin and Hippo–YAP to reinforce stem‐like states [37, 38]. Pharmacological inhibition of autophagy significantly depletes stemness populations and restores chemosensitivity. Moreover, AMP‐activated protein kinase (AMPK), a central energy sensor and positive regulator of autophagy, has emerged as a key modulator of cancer stemness through stress‐induced metabolic reprogramming [39]. Despite these insights, the molecular mechanisms by which autophagy coordinates stemness‐driven chemoresistance in PDAC remain poorly elusive, particularly regarding crosstalk between autophagy and cancer stemness.

Serine/threonine/tyrosine kinase 1 (STYK1, also known as NOK) is an atypical receptor tyrosine kinase (RTK) characterized by an intracellular kinase domain, a single transmembrane domain, and a truncated extracellular region that lacks canonical ligand‐binding capacity [24, 40]. STYK1 is aberrantly overexpressed in malignancies including colorectal [41], non‐small cell lung [42], and breast cancers [43]. Functionally, STYK1 drives tumor progression and metastasis by activating oncogenic PI3K/AKT, JAK/STAT3, and MEK/ERK cascades [44, 45]. Our previous work identified STYK1 as a critical autophagy regulator that directly binds the autophagy‐specific class III phosphatidylinositol 3‐kinase complex I (PtdIns3K‐C1), thereby enhancing BECN1 phosphorylation at Ser90 to disrupt the BECN1‐BCL2 interaction and initiate autophagic flux [24]. Recently, we further demonstrated that STYK1 directly binds GSK3β and β‐catenin, promotes sequestration of membrane‐associated GSK3β into multivesicular bodies (MVBs), which leads to hyperactivation of the Wnt/β‐catenin pathway and drives PDAC development [46]. Although emerging evidence implicates STYK1 in mediating drug resistance [47, 48, 49], its specific mechanistic role in modulating gemcitabine sensitivity in PDAC has remained unclear.

In this study, we integrate in vitro and in vivo evidence to establish STYK1 as a key driver of gemcitabine resistance in PDAC. Mechanistically, STYK1 sustains pancreatic cancer stemness in an autophagy‐dependent manner. Moreover, under gemcitabine‐induced stress, AMPK activation promotes STYK1 phosphorylation at Ser91 and Ser304, thereby further potentiating STYK1‐mediated autophagy and stemness maintenance. Importantly, we engineered a rationally designed peptide that disrupts the STYK1‐AMPK interaction. This peptide acts synergistically with gemcitabine to suppress tumor progression in preclinical models of gemcitabine‐resistant PDAC, offering a potential therapeutic strategy for this intractable malignancy.

## 2 Results

### 2.1 STYK1 Promotes Gemcitabine Resistance in Pancreatic Cancer Cell

To identify whether STYK1 regulates gemcitabine resistance and the specific mechanism involved, we first analyzed human pancreatic cancer samples from Gene Expression Omnibus (GEO) datasets (GSE46203), which revealed that gemcitabine treatment induced the up‐regulation of *STYK1* in both a dose‐ and time‐dependent manner (Figure S1A). Similar results were observed in human pancreatic cancer cells (Figures 1A,B and S1B,C). Furthermore, we established gemcitabine‐resistant pancreatic cancer cell lines (PANC‐1/GEM and AsPC‐1/GEM), both of which exhibited significantly reduced sensitivity to gemcitabine compared with pancreatic cancer cells, confirming successful model establishment (Figure S1D, E). Notably, STYK1 expression was significantly elevated in PANC‐1/GEM and AsPC‐1/GEM cells compared with PANC‐1 and AsPC‐1cells (Figure 1C,D). Importantly, IHC staining further revealed that STYK1 expression was markedly upregulated in gemcitabine‐resistant PDX tumor tissues (Figure 1E).

**FIGURE 1:**
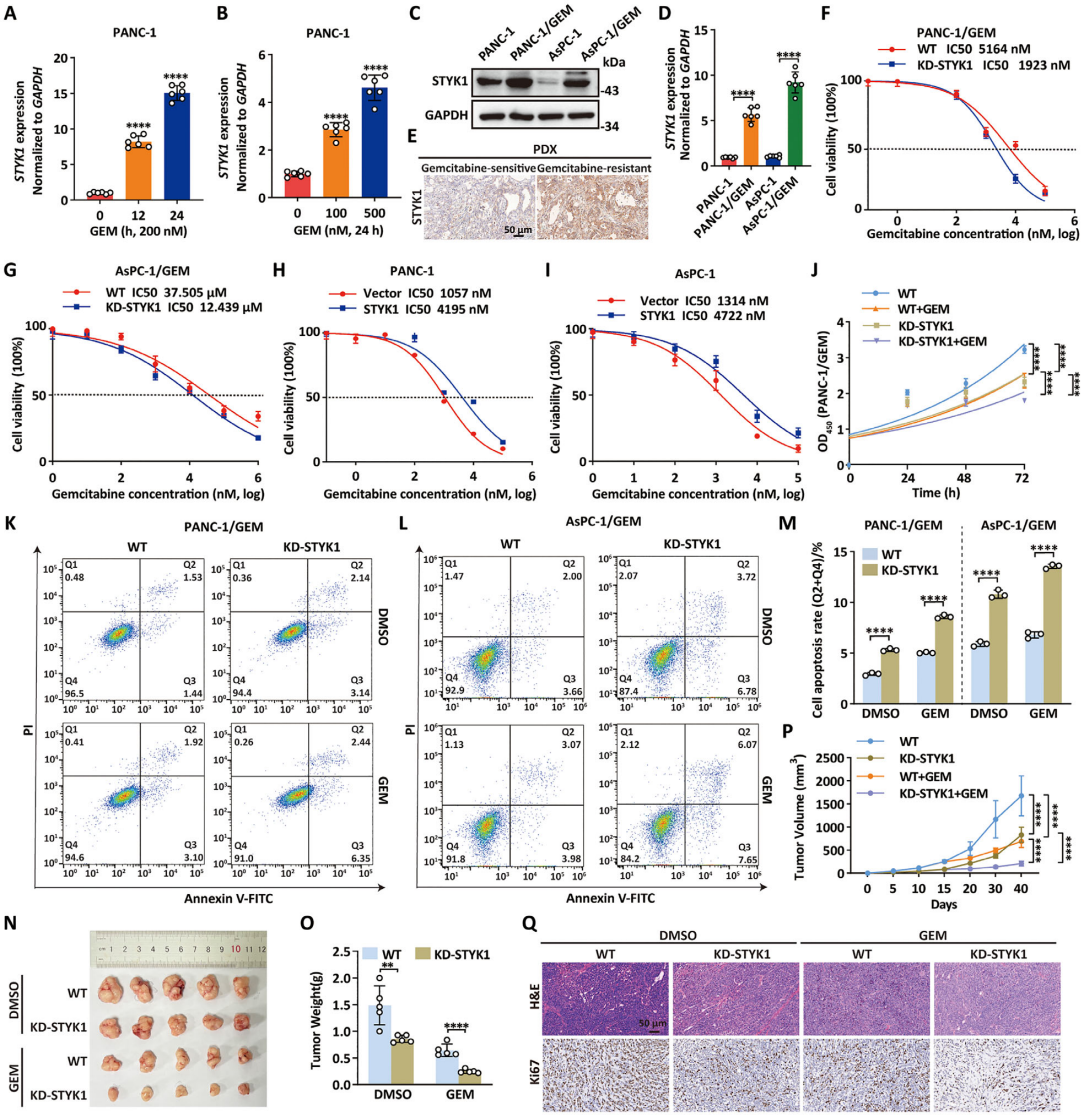
STYK1 promotes gemcitabine resistance in pancreatic cancer cells. (A, B) PANC‐1 cells were treated with different concentrations of gemcitabine for the indicated time periods, and *STYK1 mRNA* expression was assessed by RT‐qPCR (n = 6). **** *p* < 0.0001 according to Student's *t*‐test. (C) Western blotting analysis of STYK1 expression levels in human pancreatic cancer cell lines PANC‐1 and AsPC‐1, as well as in the gemcitabine‐resistant cell lines PANC‐1/GEM and AsPC‐1/GEM. D) RT‐PCR analysis of *STYK1 mRNA* levels in PANC‐1, AsPC‐1, PANC‐1/GEM and AsPC‐1/GEM cells. ****p < 0.0001 according to Student's *t*‐test. (E) Representative images of IHC staining for STYK1 in gemcitabine‐sensitive and resistant pancreatic cancer PDX tumors tissues. (F–I) Cell viability of PANC‐1/GEM and AsPC‐1/GEM cells with stable STYK1 knockdown, as well as PANC‐1 and AsPC‐1 cells stably overexpressing STYK1, following treatment with different concentrations of gemcitabine for 72 h, as determined by CCK‐8 assay. (J) PANC‐1/GEM cells with stable STYK1 knockdown were treated with gemcitabine (5 µM) for different durations as indicated, and OD values were assessed using the CCK‐8 assay. ****p < 0.0001 according to Student's *t*‐test. (K–M) Apoptosis of PANC‐1/GEM and AsPC‐1/GEM cells with stable STYK1 knockdown following gemcitabine treatment (5 µM for PANC‐1/GEM and 40 µM for AsPC‐1/GEM, 24 h), assessed using an Annexin V‐AbFluor 488/PI double staining Apoptosis Detection Kit. Annexin V‐positive cells indicate early apoptosis, whereas PI‐positive cells indicate late apoptosis/necrosis (n = 3). ****p < 0.0001 according to Student's *t*‐test. (N) Representative images of resected tumors from nude mice in different groups (n = 5). O) Tumor weights of resected tumors in different groups of mice (n = 5). ****p < 0.0001 according to Student's *t*‐test. **p < 0.01; ****p < 0.0001 according to Student's *t*‐test. (P) Tumor volume change curves in different groups of mice (n = 5); Tumor volume was measured every 5 days after cell injection and during gemcitabine treatment (25 mg/kg). ****p < 0.0001 according to Student's *t*‐test. (Q) Representative H&E staining and Ki67 immunohistochemical staining images of resected tumor tissues (n = 5). Scale bar: 50 µm. Data were expressed as mean ± SD, ^*^ *p* < 0.05; ^**^ *p* < 0.01; ^***^ *p* < 0.001; ^****^ *p* < 0.0001 according to Student's *t*‐test.

To investigate STYK1 function, we generated PANC‐1 and AsPC‐1 cells stably overexpressing STYK1, and PANC‐1/GEM and AsPC‐1/GEM cells stably STYK1 knockdown via lentiviral infection. The expression of STYK1 was increased or decreased as expected (Figure S1F,G). The KD‐STYK1#1 clone, which displayed the most efficient knockdown, was selected for subsequent experiments. Cell viability assays showed that STYK1 knockdown resensitized PANC‐1/GEM and AsPC‐1/GEM cells to gemcitabine, markedly reducing the gemcitabine IC50 value, while STYK1 overexpression induced resistance (Figure 1F–I). We further investigated whether STYK1 affects the tumorigenicity of pancreatic cancer cells inhibited by gemcitabine. The results demonstrated that STYK1 knockdown significantly promoted gemcitabine‐induced apoptosis while inhibiting proliferation, whereas STYK1 overexpression had the opposite effect (Figures 1J–M and S1H–P). Trans‐well assays further demonstrated that STYK1 promoted cell migration and invasion under gemcitabine treatment (Figure S1Q–X).

To further evaluate the effect of STYK1 on the sensitivity of pancreatic cancer to gemcitabine in vivo, subcutaneous pancreatic xenograft tumors in BALB/c nude mice were derived from PANC‐1/GEM cells with stable STYK1 knockdown. Consistent with in vitro data, STYK1 knockdown significantly reduced tumor growth and Ki67 expression upon gemcitabine treatment (Figure 1N–Q). Collectively, these data indicate that gemcitabine treatment upregulates STYK1 expression, and high STYK1 expression contributes to gemcitabine resistance in pancreatic cancer cells, both in vitro and in vivo.

### 2.2 STYK1 Facilitates Pancreatic Cancer Stemness Upon Gemcitabine Treatment by Promoting Autophagy Activation

To further elucidate the molecular basis by which STYK1 drives gemcitabine resistance in pancreatic cancer, we performed RNA sequencing (RNA‐Seq) analysis in AsPC‐1/GEM cells with stable STYK1 knockdown. Kyoto Encyclopedia of Genes and Genomes (KEGG) enrichment analysis of the differentially expressed genes revealed that STYK1 knockdown affected the regulation of stemness‐associated signaling pathways, which have been closely implicated in gemcitabine resistance in pancreatic cancer [50] (Figure 2A). Furthermore, bioinformatic interrogation of The Cancer Genome Atlas (TCGA) demonstrated positive correlations between STYK1 expression and pancreatic cancer stemness markers CD44 and CD133 (Figure S2A). Consistently, histological analysis revealed expression of CD44 and CD133 was significantly reduced in the pancreas of KPCS mice compared to KPC mice (Figure 2B). Importantly, STYK1 knockdown significantly reduced the protein and transcriptional levels of CD44 and CD133 after gemcitabine exposure (Figure 2C–E), an effect further confirmed in xenograft tumors by immunohistochemistry (Figure 2F). Functionally, an in vitro sphere formation assays further demonstrated that STYK1 knockdown significantly impaired the sphere‐forming capacity of gemcitabine‐resistant PDAC cells following gemcitabine treatment (Figure 2G). Importantly, flow cytometric analysis demonstrated that STYK1 knockdown markedly reduced the gemcitabine‐induced increase in the proportion of CD44^+^CD133^+^ cells (Figure 2H,I). Conversely, STYK1 overexpression amplified stemness phenotypes under gemcitabine stress (and Figures 2J and S2B–F), establishing STYK1 as a stemness amplifier that drives chemoresistance.

FIGURE 2STYK1 facilitates pancreatic cancer stemness upon gemcitabine treatment by promoting autophagy activation. (A) KEGG pathway enrichment analysis of differentially expressed genes after STYK1 knockdown in AsPC‐1/GEM cells. (B) Representative immunohistochemical images of pancreatic cancer stemness markers CD44 and CD133 in pancreatic tissue resected from *Styk1* knockout KPC mice. Scale bar: 40 µm. (C) Western blotting analysis of CD44, CD133 and LC3 protein expression levels in PANC‐1/GEM and AsPC‐1/GEM cells with stable STYK1 knockdown treated with or without gemcitabine (5 µM for PANC‐1/GEM cells, or 40 µµ for AsPC‐1/GEM cells, 24 h). (D, E) RT‐qPCR analysis of *CD44*, *CD133*, and stemness‐associated transcription factors *OCT4*, *NANOG*, and *SOX2* in PANC‐1/GEM and AsPC‐1/GEM cells with stable STYK1 knockdown treated with or without gemcitabine (5 µM for PANC‐1/GEM cells, or 40 µµ for AsPC‐1/GEM cells, 24 h) (n = 3). *p < 0.05; **p < 0.01; ***p < 0.001; ****p < 0.0001 according to Student's *t*‐test. (F) Representative immunohistochemical images of CD44 and CD133 in xenograft tumor tissue. Scale bar: 100 µm. (G) Representative images of AsPC‐1/GEM cells with stable STYK1 knockdown after 14 days of culture in serum‐free medium treated with or without gemcitabine (5 µM for PANC‐1/GEM cells, or 40 µµ for AsPC‐1/GEM cells). The number of spheroids with a diameter of >70 µm was statistically analyzed (n = 3). Scale bar: 100 µm. *p < 0.05; **p < 0.01 according to Student's *t*‐test. (H, I) Flow cytometric analysis and quantification of CD44^+^CD133^+^ cells in PANC‐1/GEM and AsPC‐1/GEM cells with stable STYK1 knockdown treated with or without gemcitabine (5 µM for PANC‐1/GEM cells, or 40 µµ for AsPC‐1/GEM cells, 24 h). Numbers in the graphs indicate the percentage of the subpopulation (n = 3). *p < 0.05; **p < 0.01; ****p < 0.0001 according to Student's *t*‐test. (J) Quantification of CD44^+^CD133^+^ cells in PANC‐1 and AsPC‐1 cells with stable STYK1 overexpression treated with or without gemcitabine (1 µM, 24 h). Numbers in the graphs indicate the percentage of the subpopulation (n = 3). ***p < 0.001; ****p < 0.0001 according to Student's *t*‐test. (K) Western blotting analysis of CD44, CD133, and LC3 protein expression levels in PANC‐1 and AsPC‐1 cells with stable STYK1 overexpression or/and ATG7 knockdown. (L, M) RT‐qPCR analysis of *CD44*, *CD133*, and *OCT4*, *NANOG*, *SOX2* in PANC‐1 and AsPC‐1 cells with stable STYK1 overexpression or/and ATG7 knockdown (n = 3). *p < 0.05; ns (no significance) according to Student's *t*‐test. (N, O) Representative images of AsPC‐1 cells with stable STYK1 overexpression or/and ATG7 knockdown and AsPC‐1/GEM cells with stable STYK1 knockdown treated with or without gemcitabine autophagy inhibitor CQ (10 µM, 6 h) after 14 days of culture in serum‐free medium. The number of spheroids with a diameter of >70 µm was statistically analyzed (n = 3). Scale bar: 100 µm. *p < 0.05; ns (no significance) according to Student's *t*‐test. (P, Q) Flow cytometric analysis and quantification of CD44^+^CD133^+^ cells in PANC‐1 and AsPC‐1 cells with stable STYK1 overexpression or/and ATG7 knockdown. Numbers in the graphs indicate the percentage of the subpopulation (n = 3). *p < 0.05; ***p < 0.001; ns (no significance) according to Student's *t*‐test. (R) Quantification of CD44^+^CD133^+^ cells in PANC‐1/GEM and AsPC‐1/GEM cells with stable STYK1 knockdown treated with or without autophagy inhibitor CQ (10 µM, 6 h). Numbers in the graphs indicate the percentage of the subpopulation (n = 3). *p < 0.05; ns (no significance) according to Student's *t*‐test. Data were expressed as mean ± SD.

**Figure:**
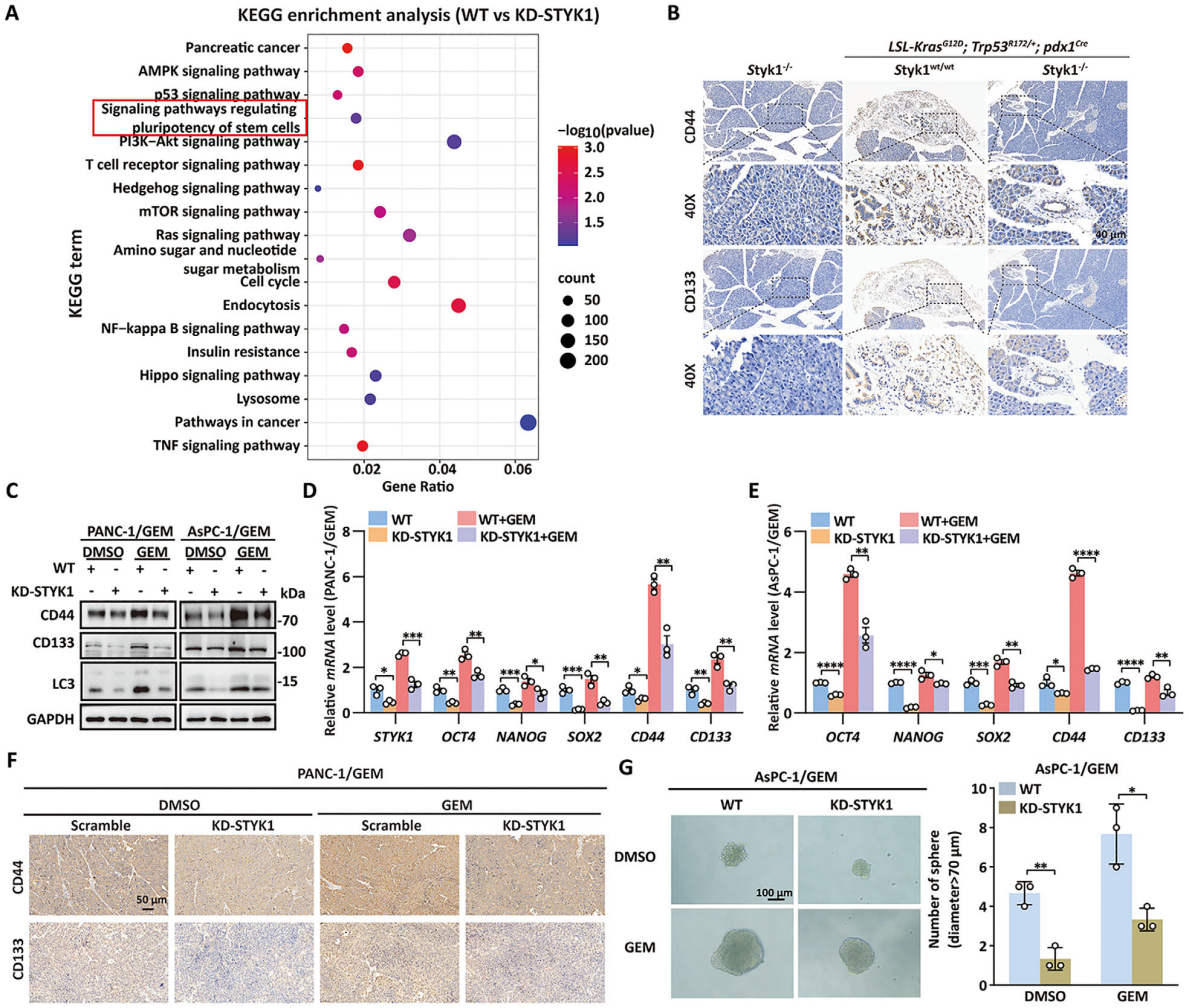

**Figure:**
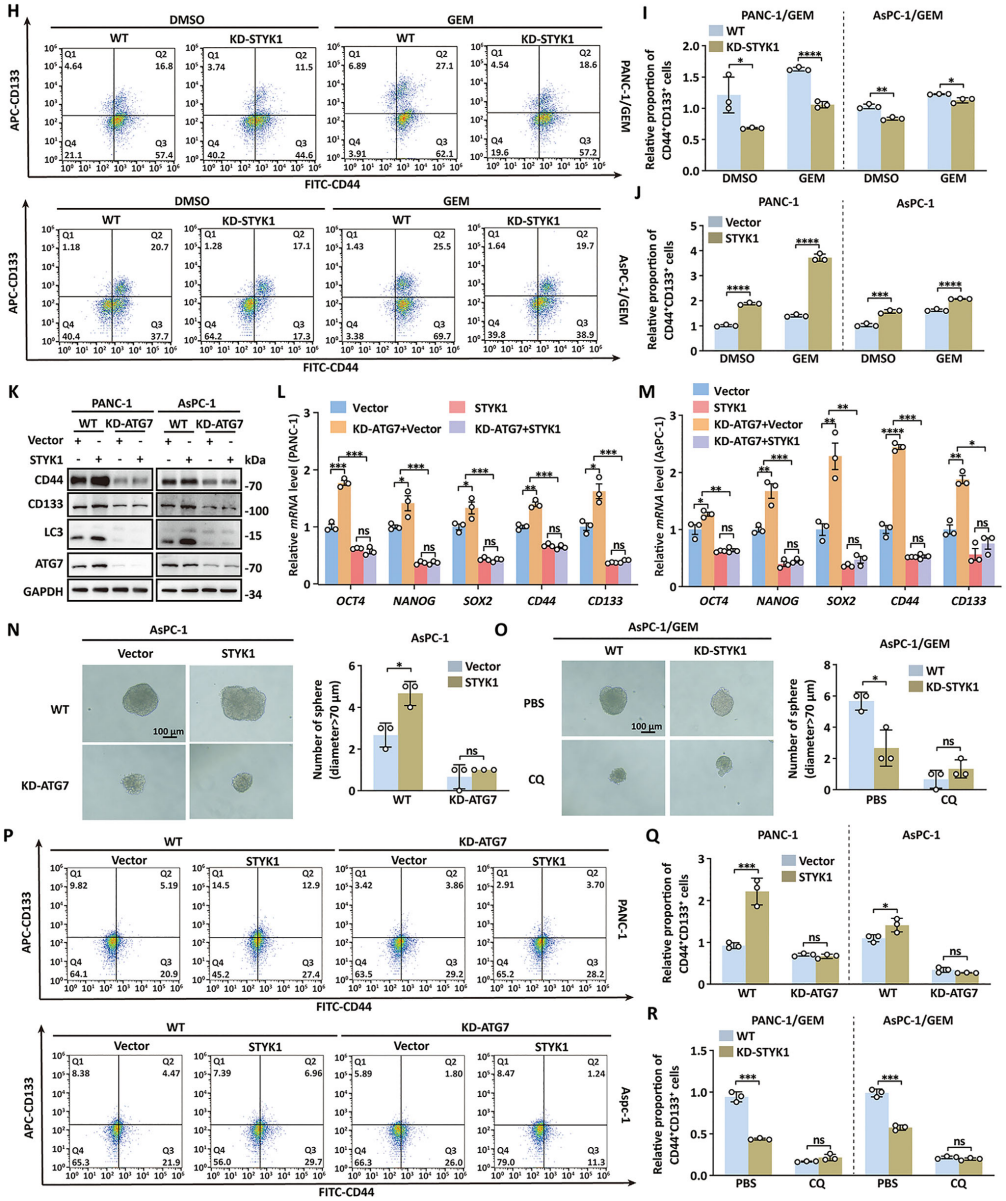

Given that STYK1 has been shown to initiate autophagy by directly binding to the PI3KC1 complex, and considering the critical role of autophagy in sustaining pancreatic cancer stemness under chemotherapeutic stress [23, 45], we next investigate whether STYK1 regulates stemness via autophagy. It was found that inhibition of autophagy by either KD‐ATG7 or CQ abrogated STYK1‐induced upregulation of CD44 and CD133 (Figures 2K–M and S2G–I), as well as tumor sphere formation and the proportion of CD44^+^CD133^+^ cells (Figures 2N–R and S2J). Collectively, these findings demonstrate that gemcitabine treatment induces STYK1‐mediated autophagy activation to promote pancreatic cancer stemness, thereby contributing to chemoresistance.

### 2.3 Gemcitabine‐Induced AMPK to Amplify STYK1‐Mediated Autophagy Activation

To explore the mechanism by which gemcitabine further promotes STYK1‐mediated autophagy activation, we focused on the AMPK signaling pathway, a key regulator of autophagy that responds to gemcitabine‐induced cellular stress and is closely associated with gemcitabine‐induced resistance in pancreatic cancer [51]. The results showed that gemcitabine treatment dose‐and time‐dependent autophagic flux enhancement, accompanied by AMPK activation in PANC‐1/GEM cells (Figures 3A–D and S3A–D). KEGG enrichment analysis (GSE80617) confirmed AMPK signaling enrichment in gemcitabine resistance pathways (Figure 3E). Next, we investigated the role of AMPK in STYK1‐mediated autophagy regulation. Our results revealed that inhibition of AMPK using Compound C exacerbated LC3‐II reduction in STYK1‐knockdown cells, whereas AMPK activation via EBSS starvation or AICAR treatment rescued autophagy suppression (Figure S3E–H). Consistently, gemcitabine‐induced AMPK activation promoted STYK1‐dependent autophagic flux, while AMPK deficiency or Compound C treatment attenuated gemcitabine‐induced LC3‐II accumulation (Figure 3F–I). Given the function of STYK1 in facilitating PI3KC1 complex assembly (ATG14L‐Beclin1‐VPS34), we assessed the effect of AMPK activation on this process. Our findings showed that both gemcitabine‐ and AICAR‐induced AMPK activation further enhanced STYK1‐mediated ATG14L‐Beclin1‐VPS34 complex formation (Figures 3J,K and S3I). These results suggest that gemcitabine‐triggered AMPK activation amplifies STYK1‐mediated autophagy initiation through potentiation of PI3KC1 complex assembly.

**FIGURE 3:**
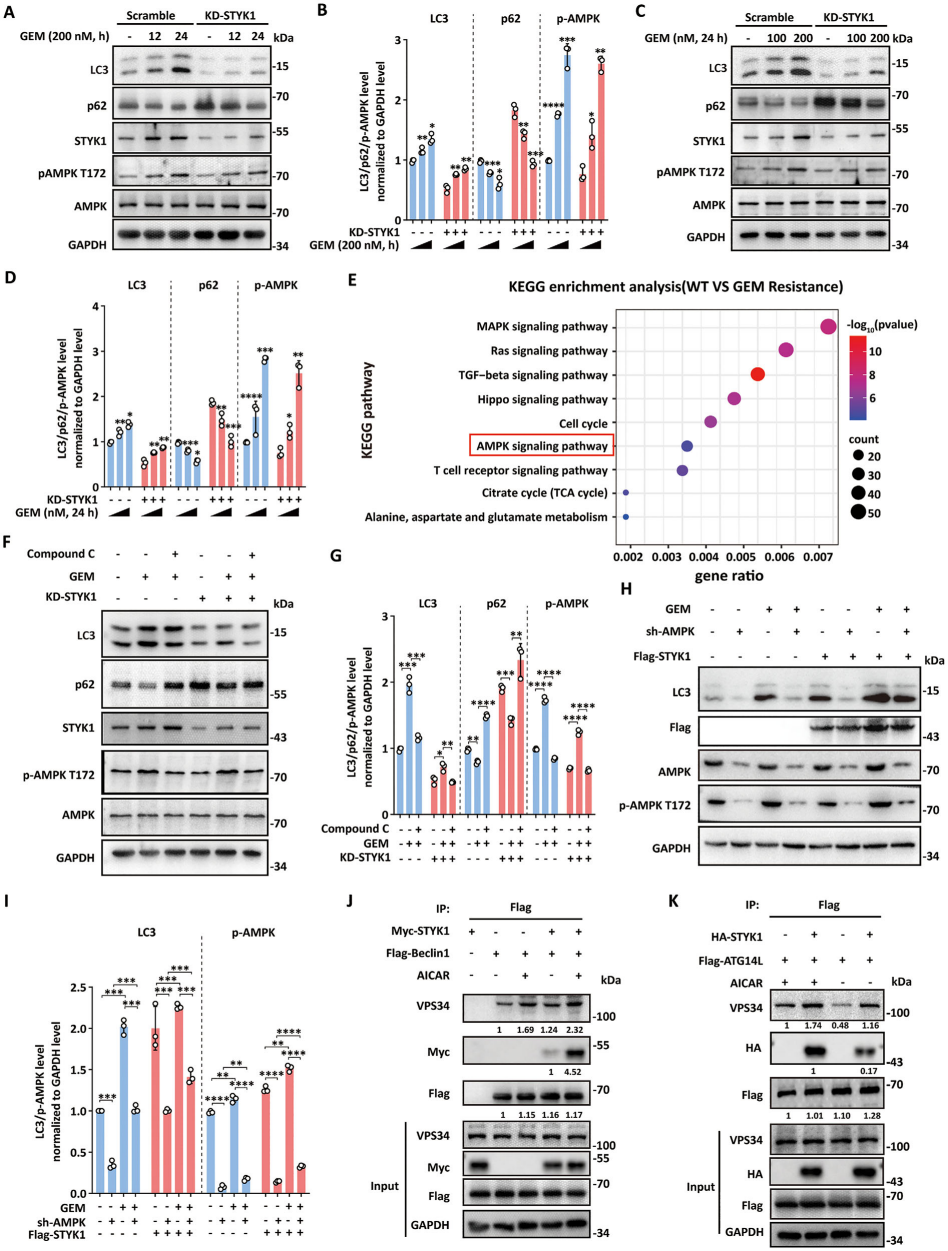
Gemcitabine induced AMPK to amplify STYK1‐mediated autophagy activation. (A–D) Western blotting analysis and quantification of LC3, p62, STYK1, and p‐AMPK T172 protein expression levels in PANC‐1/GEM cells with wildtype and stable STYK1 knockdown, treated with gemcitabine at different concentrations and for different durations (n = 3). *p < 0.05; **p < 0.01; ***p < 0.001; ****p < 0.0001 according to Student's *t*‐test. (E) KEGG enrichment analysis of differentially expressed genes between gemcitabine‐resistant pancreatic cancer cells and pancreatic cancer cells (GSE80617). Differentially expressed genes were identified using the criteria of |log2FC| > 1 and *p* < 0.05. The dot size represents the number of genes enriched in each pathway, and the color represents the enrichment significance based on −log10(*p* value). (F, G) Western blotting analysis and quantification of LC3, p62, and p‐AMPK T172 protein expression levels in PANC‐1/GEM cells with wildtype and stable STYK1 knockdown treated with or without the AMPK inhibitor Compound C (10 µM, 24 h) or gemcitabine (5 µM, 24 h) (n = 3). *p < 0.05; **p < 0.01; ***p < 0.001; ****p < 0.0001 according to Student's *t*‐test. (H, I) Western blotting analysis and quantification of LC3 and p‐AMPK T172 protein expression levels in AMPK‐deficient PANC‐1 cells transfected with Flag‐STYK1 and treated with or without gemcitabine (1 µM, 24 h) (n = 3). **p < 0.01; ***p < 0.001; ****p < 0.0001 according to Student's *t*‐test. (J, K) Co‐immunoprecipitation analysis of the interaction between STYK1 and VPS34 with Beclin1 or ATG14L in HEK293T cells co‐transfected with STYK1 and Flag‐Beclin1 or Flag‐ATG14L, treated with or without treatment AMPK activator AICAR (1 mM, 4 h). Cell lysates were used for IP and Western blotting with the indicated antibodies. Data were expressed as mean ± SD, *p < 0.05; **p < 0.01; ***p < 0.001; ****p < 0.0001 according to Student's *t*‐test.

### 2.4 AMPK Directly Binds and Phosphorylates STYK1 at S91 and S304

To explore the mechanism underlying AMPK‐enhanced STYK1‐mediated autophagy activation, we performed Flag‐tag affinity purification of STYK1‐containing complexes in HEK293T cells, revealing a robust interaction between AMPK and STYK1 (Figure 4A). This interaction was confirmed by co‐immunoprecipitation (co‐IP) and immunofluorescence colocalization (Figures 4B,C and S4A). To further validate binding specificity, we expressed recombinant His‐tagged truncated N‐terminal exo‐structural domains (1‐26aa) and transmembrane structural domains (27‐46aa) of STYK1 (designated as His‐STYK1‐ICD) as well as GST‐tagged AMPK in a prokaryotic bacterial expression system. GST pull‐down assays demonstrated that purified His‐STYK1‐ICD bound directly to GST‐AMPK (Figure 4D). We next mapped the AMPK‐binding domain using five STYK1 truncation mutants. Co‐IP analysis identified the kinase domain (117‐378aa) as essential for AMPK binding (Figure 4E). Notably, AMPK activation by gemcitabine, EBSS starvation, or AICAR treatment significantly strengthened the STYK1‐AMPK interaction (Figures 4F and S4B,C).

**FIGURE 4:**
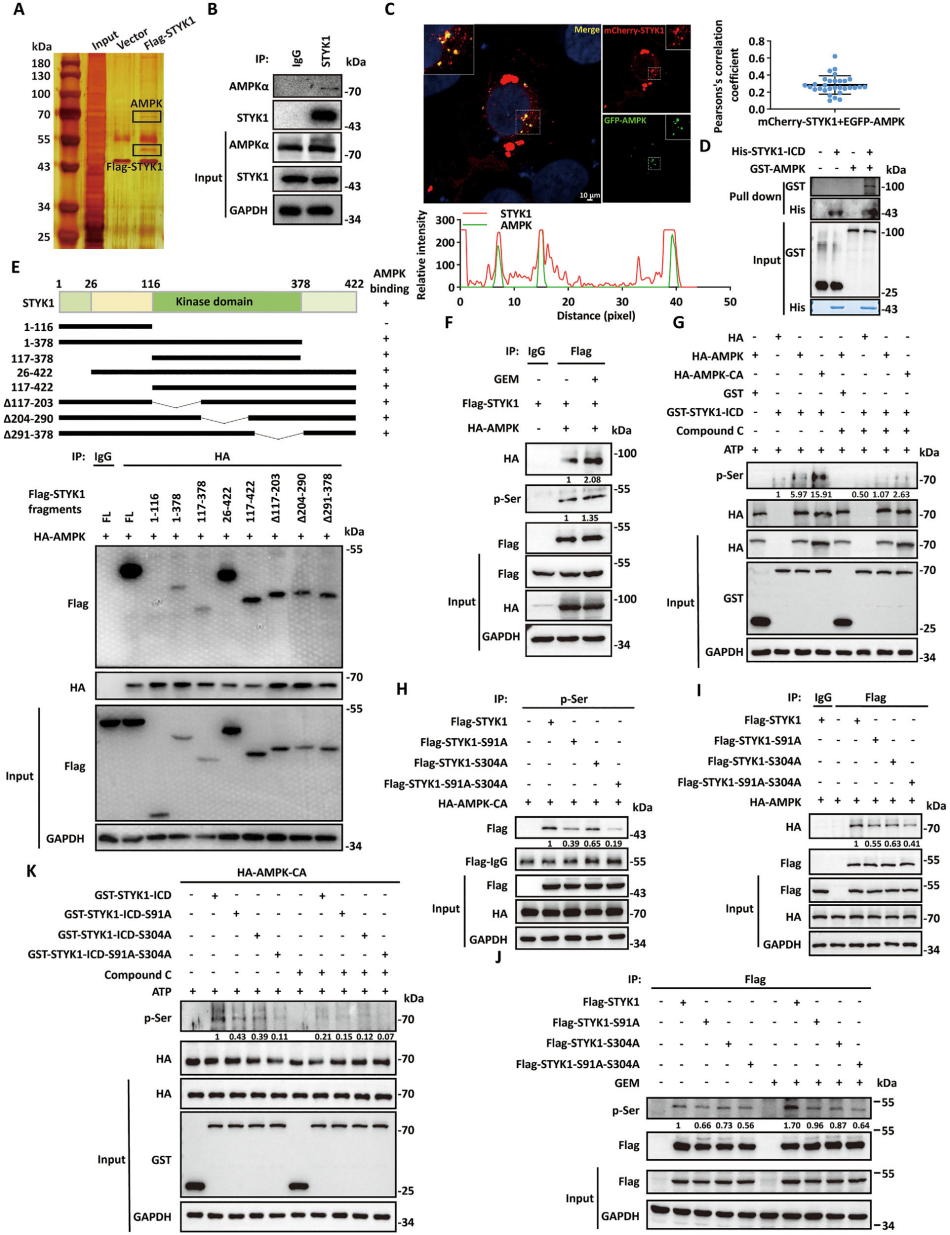
AMPK directly binds and phosphorylates STYK1 at S91 and S304. (A) Silver staining of proteins immunoprecipitated with Flag antibody from HEK293T cells transfected with Flag‐tagged STYK1. (B) Co‐immunoprecipitation analysis of the interaction between endogenous STYK1 and AMPK in PANC‐1 cells. Cell lysates were used for IP and Western blotting with the indicated antibodies. (C) Confocal microscopy analysis and Pearson's correlation quantification of the co‐localization between mCherry‐tagged STYK1 and EGFP‐tagged AMPK in Hela cells (n = 30). Plot profile analysis of protein colocalization. The X‐axis represents the distance in pixels along the region of interest, and the Y‐axis indicates the relative fluorescence intensity of each channel. Scale bar: 10 µm. (D) Externally purified recombinant GST‐tagged STYK1 intracellular domain lacking the N‐terminal extracellular domain (aa 1–26) and transmembrane domain (aa 27–46), referred to as STYK1‐ICD, was incubated with purified recombinant His‐tagged AMPK, mixture was pulled down by GST beads, and then the precipitated proteins were analyzed by Western blotting. (E) HEK293T cells were co‐transfected with HA‐AMPK and Flag‐tagged wild‐type or mutant STYK1 constructs for 48 h and then harvested for IP and Western blotting analysis. The schematic diagram of STYK1 truncation generation and the AMPK‐STYK1 interaction domains is shown. (F) Co‐immunoprecipitation analysis of the interaction between STYK1 and AMPK, and total serine phosphorylation of STYK1 in HEK293T cells treated with or without gemcitabine (5 µM, 24 h). Cell lysates were used for IP and Western blotting with the indicated antibodies. (G) AMPK kinase was obtained by immunoprecipitation from lysates of HEK293T cells transfected with HA‐tagged wild‐type AMPK or the constitutively active AMPK mutant AMPK‐CA (T172D). The immunoprecipitated AMPK kinase was then incubated with purified recombinant GST‐tagged STYK1‐ICD in the presence or absence of the AMPK inhibitor Compound C at 30°C for 30 min, followed by Western blotting analysis. (H–J) Total serine phosphorylation levels of STYK1 and its mutants, as well as their interactions with AMPK, were evaluated in HEK293T cells. Cells were co‐transfected with Flag‐tagged wild‐type or mutant STYK1 constructs and HA‐tagged AMPK or constitutively active AMPK‐CA plasmids, followed by treatment with or without gemcitabine (5 µM, 24 h). Cell lysates were used for IP and Western blotting with the indicated antibodies. K) Total serine phosphorylation levels of purified GST‐STYK1‐ICD and its mutants under conditions of AMPK inhibitor compound C after incubation with HA‐AMPK‐CA (T172D) obtained from HEK293T cell lysates.

Building on our previous discovery that EGFR‐TKI‐induced AMPK activation phosphorylates STYK1 at Ser304 [52]. Here, we demonstrated that AMPK activation by gemcitabine or EBSS similarly enhances serine phosphorylation of STYK1 (Figures 4F and S4D). Notably, in vitro kinase assays showed that AMPK directly phosphorylates recombinant GST‐STYK1‐ICD in a kinase activity‐dependent manner (Figure 4G). Next, building on the AMPK consensus motif ([M/V/L/I/F][R/K/H]XX[pS/pT]XXX[M/V/L/I/F]) from Human Protein Reference Database [53], we identified Ser91 within the conserved “LKETS(91)VENF” sequence as a novel potential phosphorylation site in human STYK1. To validate direct AMPK‐dependent phosphorylation at Ser91 and Ser304, we generated STYK1 phospho‐deficient mutants (S91A, S304A, and the S91A‐S304A double mutant). We found that both the S91A and S304A mutations significantly attenuated AMPK CA‐mediated serine phosphorylation of STYK1 (Figures 4H and S4E) and weakened the STYK1‐AMPK interaction (Figure 4I). Moreover, STYK1 S91A and S304A mutants significantly reduced gemcitabine induced serine phosphorylation of STYK1 (Figure 4J). Conversely, AMPK depletion significantly attenuated phosphorylation at both S91 and S304 residues (Figure S4F). Consistent results were obtained in an in vitro phosphorylation assay using purified bacterially expressed GST‐STYK1‐ICD and immunoprecipitated AMPK‐CA (Figures 4K and S4G). Collectively, these data demonstrate that AMPK‐mediated phosphorylation of STYK1 at S91 and S304 may stabilize or enhance the STYK1‐AMPK interaction.

### 2.5 Phosphorylated STYK1 Future Promotes Autophagy, Cancer Stemness and Pancreatic Cancer Progression

To investigate the role of AMPK‐mediated STYK1 phosphorylation in autophagy, we first assessed LC3‐II levels in PANC‐1 cells expressing STYK1 phospho‐deficient mutants. The results showed that AICAR‐induced AMPK activation partially alleviated the impairment of autophagic flux observed in cells expressing STYK1 mutants (Figure 5A,B). Critically, AMPK depletion further intensified this flux suppression under gemcitabine treatment (Figure 5C,D), with CQ blockade yielding consistent effects (Figure S5A,B). Furthermore, GFP‐ZFYVE1 and GFP‐WIPI1 revealed impaired autophagosome formation in mutants (Figure S5C,D). Consistently, mRFP‐GFP‐LC3 tandem reporter gene assays demonstrated that STYK1 mutants significantly reduced both autophagosomes (GFP‐positive/RFP‐positive, yellow puncta) and autolysosomes (GFP‐negative/RFP‐positive, red puncta) formation after gemcitabine treatment (Figure 5E,F).

**FIGURE 5:**
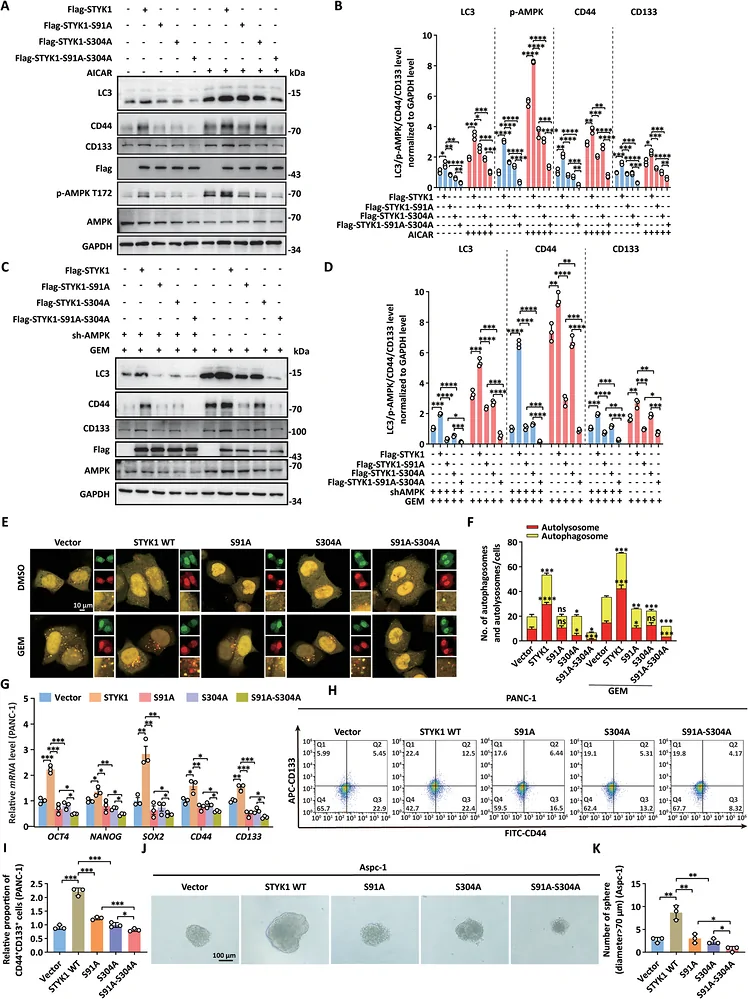
Phosphorylated STYK1 further promotes autophagy, cancer stemness, and pancreatic cancer progression. (A, B) Western blotting analysis and quantification of LC3, CD44, CD133, and p‐AMPK T172 protein expression levels in PANC‐1 cells expressing Flag‐tagged wild‐type or mutant STYK1 constructs. Cells were treated with or without the AMPK activator AICAR (1 mM, 4 h) (n = 3). *p < 0.05; **p < 0.01; ***p < 0.001; ****p < 0.0001 according to Student's *t*‐test. (C, D) Western blotting analysis and quantification of LC3, CD44, and CD133 protein expression levels in PANC‐1 cells expressing Flag‐tagged wild‐type or mutant STYK1 constructs under AMPK depletion. Cells were treated with or without gemcitabine (1 µM, 24 h) (n = 3). *p < 0.05; **p < 0.01; ***p < 0.001; ****p < 0.0001 according to Student's *t*‐test. (E, F) Representative confocal images of red and yellow spots in PANC‐1 cells stably overexpressing STYK1 and its mutants, treated with or without gemcitabine (1 µM, 24 h). Quantitative measurement of the number of autolysosomes (red spots) and autophagosomes (yellow spots) (n = 3). Scale bar: 10 µm. *p < 0.05; **p < 0.01; ***p < 0.001; ****p < 0.0001; ns (no significance) according to Student's *t*‐test. (G) RT‐qPCR analysis of *CD44*, *CD133*, and stemness‐associated transcription factors *OCT4*, *NANOG*, and *SOX2* in PANC‐1 cells stably overexpressing STYK1 and its mutants (n = 3). *p < 0.05; **p < 0.01; ***p < 0.001 according to Student's *t*‐test. (H, I) Flow cytometric analysis and quantification of CD44^+^CD133^+^ cells in PANC‐1 cells stably overexpressing STYK1 and its mutants. Numbers in the graphs indicate the percentage of the subpopulation (n = 3). *p < 0.05; ***p < 0.001 according to Student's *t*‐test. J, K) Representative images of AsPC‐1 cells stably overexpressing STYK1 and its mutants cultured in serum‐free medium for 14 days. The number of spheroids with a diameter of >70 µm was statistically analyzed (n = 3). Scale bar: 100 µm. *p < 0.05; **p < 0.01 according to Student's *t*‐test. Data were expressed as mean ± SD.

We next assessed the role of STYK1 phosphorylation in pancreatic cancer stemness. The results showed that, compared with the control, treatment with the AMPK activator AICAR promoted CD44 and CD133 expression, whereas AMPK depletion further diminished CD44 and CD133 levels in phospho‐deficient mutants (Figure 5A–D). Consistently, these mutants significantly downregulated *mRNA* levels of *CD44* and *CD133*, reduced the proportion of CD44^+^CD133^+^ cells, and inhibited tumor sphere formation (Figures 5G–K and S5F–H). These results suggest that AMPK‐mediated STYK1 phosphorylation is crucial for maintaining autophagy‐mediated pancreatic cancer stemness.

To evaluate the contribution of STYK1 phosphorylation in pancreatic cancer progression, we generated PANC‐1 and AsPC‐1 cells expressing wild‐type STYK1 and phospho‐deficient mutants. Trans‐well and wound healing assays demonstrated that STYK1 mutants significantly inhibited migration and invasion of PANC‐1 and AsPC‐1 cells in vitro (Figure S5I–P). Additionally, these mutants significantly attenuated the proliferation of PANC‐1 and AsPC‐1 cells (Figure S5Q–V). These results suggest that AMPK‐mediated phosphorylation of STYK1 at S91 and S304 further promotes the proliferation, metastasis and invasion of pancreatic cancer cells.

### 2.6 Peptide Disrupting STYK1‐AMPK Interaction Inhibits Autophagy and Pancreatic Cancer Stemness

To mechanistically dissect the STYK1‐AMPK interaction governing autophagy and pancreatic cancer stemness, we designed inhibitory peptides targeting this interaction. In a previous study, we predicted the 9 α‐helices of the structural domain of STYK1 protein kinase based on the TASSER server and fused the 9 α‐helices to GFP via a flexible linker ((GGGGS)n) [46]. Screening revealed SAIDP5, with the amino acid sequence RADVWSFGILLYEMV, as the primary disruptor of STYK1‐AMPK binding (Figure 6A). Fusing SAIDP5 to the TAT cell‐penetrating peptide yielded CP‐SAIDP5, which exhibited efficient cellular internalization confirmed by FITC‐based tracking (Figure S6A). Surface plasmon resonance (SPR) verified direct interaction between CP‐SAIDP5 and AMPK (Figure 6B). Co‐IP assays showed that CP‐SAIDP5 effectively blocked the interaction between STYK1 and AMPK (Figure 6C).

**FIGURE 6:**
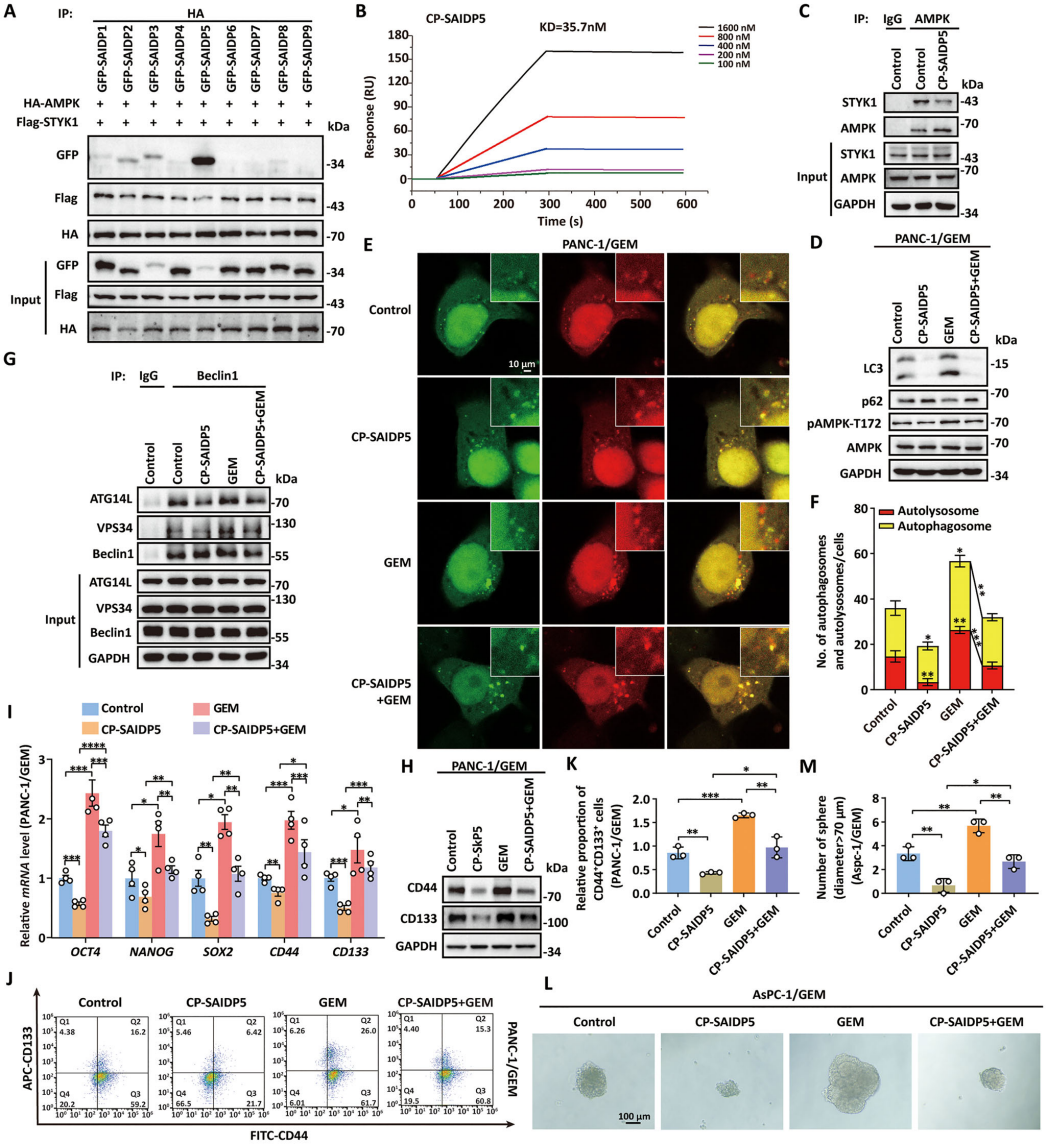
Peptide disrupting STYK1‐AMPK interaction inhibits autophagy and pancreatic cancer stemness. (A) Co‐immunoprecipitation analysis of the interaction between HA‐AMPK and GFP‐tagged STYK‐driving peptide or Flag‐STYK1 in HEK293T cells. Cell lysates were used for IP and Western blotting with the indicated antibodies. (B) SPR analysis of the binding kinetics between the peptide CP‐SAIDP5 and AMPK. (C) Co‐immunoprecipitation analysis of endogenous STYK1 and AMPK in PANC‐1/GEM cells after treatment with STYK1‐driven peptide CP‐SAIDP5 (5 µM, 24 h). Cell lysates were used for IP and Western blotting with the indicated antibodies. (D) Western blotting analysis of LC3, p62, and p‐AMPK T172 expression in PANC‐1/GEM cells after treatment with peptide CP‐SAIDP5 (5 µM, 24 h) or gemcitabine (5 µM, 24 h) alone or in combination. (E, F) Representative confocal images of red and yellow spots in PANC‐1/GEM cells after treatment with peptide CP‐SAIDP5 (5 µM, 24 h) or gemcitabine (5 µM, 24 h) alone or in combination. Quantitative measurement of the number of autolysosomes (red dots) and autophagosomes (yellow dots) (n = 3). Scale bar: 10 µm. *p < 0.05; **p < 0.01; ***p < 0.001 according to Student's *t*‐test. (G) Co‐immunoprecipitation analysis of the interaction between Beclin1 and ATG14L and VPS34 after treatment of PANC‐1/GEM cells with peptide CP‐SAIDP5 (5 µM, 24 h) or gemcitabine (5 µM, 24 h) alone or in combination. Cell lysates were used for IP and Western blotting with the indicated antibodies. (H) Western blotting analysis of CD44 and CD133 expression levels after treatment of PANC‐1/GEM cells with peptide CP‐SAIDP5 (5 µM, 24 h) or gemcitabine (5 µM, 24 h) alone or in combination. (I) RT‐qPCR analysis of *CD44*, *CD133*, and stemness‐associated transcription factors *OCT4*, *NANOG*, and *SOX2* after treatment of PANC‐1/GEM cells with peptide CP‐SAIDP5 (5 µM, 24 h) or gemcitabine (5 µM, 24 h) alone or in combination (n = 4). *p < 0.05; **p < 0.01; ***p < 0.001; ****p < 0.0001 according to Student's *t*‐test. (J, K) Flow cytometric analysis and quantification of CD44^+^CD133^+^ cells in PANC‐1/GEM cells treated with peptide CP‐SAIDP5 (5 µM, 24 h) or gemcitabine (5 µM, 24 h) alone or in combination. Numbers in the graphs indicate the percentage of this subpopulation (n = 3). *p < 0.05; **p < 0.01; ***p < 0.001 according to Student's *t*‐test. (L, M) Representative images of AsPC‐1/GEM cells treated with peptide CP‐SAIDP5 (5 µM, 24 h) or gemcitabine (5 µM, 24 h) alone or in combination, cultured in serum‐free medium for 14 days. The number of spheroids >70 µm in diameter was statistically analyzed (n = 3). Scale bar: 100 µm. *p < 0.05; **p < 0.01 according to Student's *t*‐test. Data were expressed as mean ± SD.

Next, we evaluated the impact of CP‐SAIDP5 on autophagy. The results showed that CP‐SAIDP5 significantly attenuated LC3II expression and AMPK activity (Figure 6D), while GFP‐WIPI1 and GFP‐ZFYVE1 confirmed reduced autophagosome formation (Figure S6B,C). The mRFP‐GFP‐LC3 tandem reporter gene showed that CP‐SAIDP5 reduced the number of autophagosomes and autolysosomes (Figure 6E,F), concomitant with disrupted Beclin1‐ATG14L‐VPS34 complex assembly (Figure 6G). In addition, we examined the effect of CP‐SAIDP5 on pancreatic cancer stemness. The results showed that protein and *mRNA* levels of CD44 and CD133 were reduced after CP‐SAIDP5 treatment (Figures 6H,I and S6F,G). Similarly, CP‐SAIDP5 reduced the proportion of CD44^+^CD133^+^ cells and tumor sphere formation (Figures 6J–M and S6H,I). Furthermore, CP‐SAIDP5 reversed gemcitabine‐induced autophagy and pancreatic cancer stemness (Figure 6). These data suggest that CP‐SAIDP5 inhibits autophagy and pancreatic cancer stemness by disrupting the interaction between STYK1 and AMPK, supporting this interaction as a potential therapeutic strategy in PDAC.

### 2.7 STYK1‐Driving Peptides Inhibit Gemcitabine‐Resistant Pancreatic Cancer Development

Given the established role of STYK1 in gemcitabine resistance, we next evaluated the anti‐tumor efficacy of disrupting the STYK1‐AMPK interaction in gemcitabine‐resistant PDAC cells. Combined treatment with CP‐SAIDP5 and gemcitabine synergistically suppressed the proliferation PANC‐1/GEM and AsPC‐1/GEM cells (Figure S7A–F) and markedly inhibited their migratory and invasive capabilities (Figures 7A,B and S7G–J). To further assess the in vivo therapeutic potential, gemcitabine‐resistant PDX models were treated with CP‐SAIDP5, gemcitabine, or their combination. Although either monotherapy restrained tumor growth, the combined treatment elicited a substantially stronger antitumor effect, without significant toxicity to body weight or liver and kidney function in mice, and accompanied by decreased Ki67 staining in tumor tissues (Figures 7C–G and S7K–P). Similar findings were observed in subcutaneous xenograft tumor models generated using PANC‐1/GEM cells implanted into BALB/c nude mice (Figure S7Q–U). Moreover, CP‐SAIDP5 also significantly reduced the expression of CD44, CD133, LC3 and promoted the expression of p62 in tumor tissues (Figures 7G and S7U).

**FIGURE 7:**
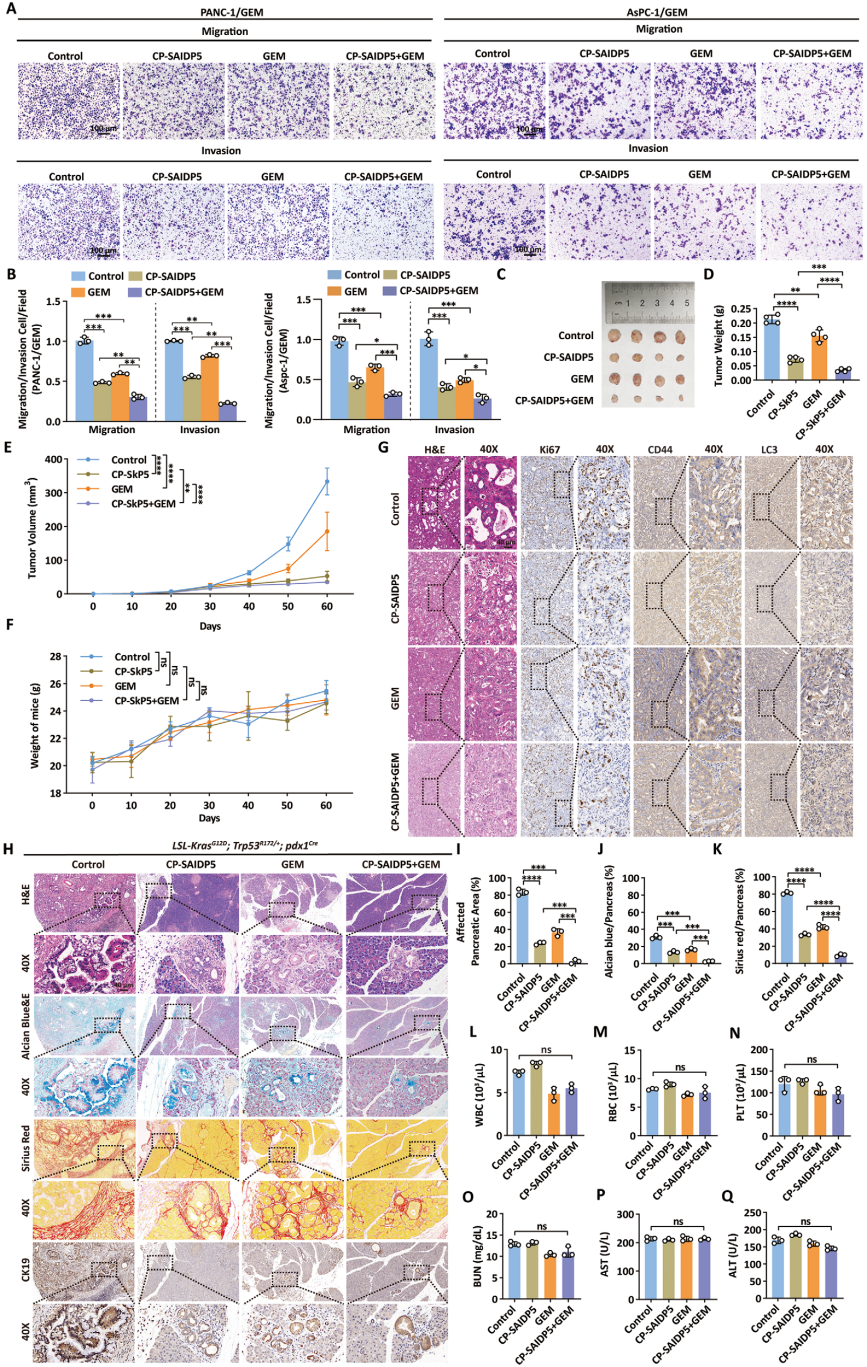
STYK1‐driving peptides inhibit gemcitabine‐resistant pancreatic cancer development. (A, B) Representative images and quantitative analysis of Transwell assays with and without matrigel of PANC‐1/GEM and AsPC‐1/GEM cells treated with peptide CP‐SAIDP5 (5 µM, 24 h) or gemcitabine (5 µM for PANC‐1/GEM cells, or 40 µµ for AsPC‐1/GEM cells, 24 h) alone or in combination (n = 3). Scale bar: 100 µm. *p < 0.05; **p < 0.01; ***p < 0.001 according to Student's *t*‐test. (C) Representative images of excised tumors from gemcitabine‐resistant PDX tumor‐bearing mice treated with CP‐SAIDP5 peptide (25 mg/kg), gemcitabine (25 mg/kg), or their combination (n = 4). (D) Tumor weights of excised PDX tumors from the indicated treatment groups (n = 4). **p < 0.01; ***p < 0.001; ****p < 0.0001 according to Student's *t*‐test. (E) Tumor growth curves of PDX‐bearing mice in the indicated treatment groups (n = 4). **p < 0.01; ****p < 0.0001 according to Student's *t*‐test. (F) Body weight changes in gemcitabine‐resistant PDX tumor‐bearing mice following treatment with CP‐SAIDP5 peptide (25 mg/kg), gemcitabine (25 mg/kg), or combination therapy. (n = 4). ns (not significant) according to Student's *t*‐test. (G) Representative H&E staining images and immunohistochemistry images of Ki67, CD44, CD133, LC3, and p62 expressed in resected tumor tissues (n = 4). Scale bar: 50 µm. (H) Representative histological images and H&E staining images of pancreatic tissues after peptide CP‐SAIDP5 (25 mg/kg) or gemcitabine (25 mg/kg) alone or co‐treated KPC mice (n = 3). Scale bar: 40 µm. I–K) Quantification of H&E staining, Sirius Red or Alcian blue staining in the indicated groups (n = 3). ***p < 0.001; ****p < 0.0001 according to Student's *t*‐test. (L–Q) Concentrations of WBC, RBC, PLT, BUN, AST, ALT in the serum of peptide CP‐SAIDP5 or gemcitabine alone or co‐treated KPC mice (n = 3). ns (not significant) according to Student's *t*‐test. Data were expressed as mean ± SD.

Given that KPC mice exhibited a similar development to human pancreatic cancer and developed invasive disease at age 14 to 16 weeks [54], we treated 8‐week‐old KPC mice with gemcitabine, CP‐SAIDP5, or both. Sirius Red/Alcian Blue staining revealed that CP‐SAIDP5 synergized with gemcitabine to reduce collagen/mucin deposition, which are key extracellular matrix components promoting tumor progression. Histopathological analysis showed significant attenuation of pancreatic intraepithelial neoplasia (PanIN), as well as the area of positive staining for Alcian Blue and Sirius Red, and positive lesions for the ductal epithelial marker cytokeratin 19 (CK19) compared to monotherapies (Figure 7H–K). Serum examination of treated KPC mice revealed no abnormalities in liver and kidney function (Figure 7L–Q), indicating that CP‐SAIDP5 does not exhibit in vivo toxicity. These findings indicate that CP‐SAIDP5 enhances gemcitabine efficacy and inhibits the progression of gemcitabine‐resistant pancreatic cancer, providing a potential combinatorial strategy against advanced pancreatic cancer.

## 3 Discussion

Gemcitabine remains a first‐line chemotherapeutic regimen for advanced PDAC, but rapid development of chemoresistance severely limits its clinical efficacy. Although both autophagy and pancreatic cancer stemness have been independently implicated in mediating drug resistance in PDAC [50, 55], the mechanistic crosstalk between these pathways in the context of gemcitabine resistance remains largely unresolved. In this study, we identify STYK1 as a critical regulator that is markedly upregulated in gemcitabine‐resistant PDAC and promotes chemoresistance by activating autophagy to sustain pancreatic cancer stemness. Importantly, AMPK‐mediated phosphorylation of STYK1 at S91 and S304 residues further amplifies STYK1‐dependent autophagy and stemness maintenance, thereby supporting gemcitabine‐resistant phenotypes in PDAC cells. Disruption of the STYK1‐AMPK interaction markedly impairs the oncogenic function of STYK1 and partially restores gemcitabine sensitivity in preclinical PDAC models (Figure S8).

During therapeutic intervention, tumor cells deploy intrinsic and adaptive mechanisms to evade drug‐induced cytotoxicity, ultimately leading to treatment failure [56, 57]. Multiple processes contribute to cancer drug resistance, including EMT, altered drug efflux, dysregulation of ATP‐binding cassette (ABC) transporters, acquired somatic mutations, and crucially, the persistence of cancer stemness properties []. Cancer cells with stemness capacity to enter a metabolically quiescent state with low proliferative activity enables them to evade drug‐induced apoptosis and survive within protective tumor microenvironments. Through a series of in vitro and in vivo experiments, we establish that STYK1 plays a critical role in mediating gemcitabine resistance in PDAC. RNA‐seq and GEPIA2 correlation analyses further indicated that STYK1 is positively associated with stemness‐related pathways, supporting a functional role for STYK1 in reinforcing stem‐like phenotypes under chemotherapeutic stress.

Autophagy serves as a critical regulator of cancer stemness, and pharmacological inhibitors chloroquine (CQ) or 3‐methyladenine (3‐MA), as well as genetic silencing of autophagy‐related genes such as ATG7 or BECN1, has been shown to deplete stemness‐sustaining populations and downregulate key pancreatic cancer stemness markers, including CD133, CD44, and ALDH1 [23, 59]. Consistently, elevated expression of autophagy markers such as ATG5, ATG7, and ATG12 directly correlates with stemness enrichment in breast and pancreatic malignancies [23, 60]. Consistent with these findings, we demonstrate that either ATG7 knockdown or CQ treatment abolishes STYK1‐mediated enhancement of stemness properties in PDAC. Crucially, the rationally designed STYK1‐targeting peptide CP‐SAIDP5 simultaneously disrupts autophagic flux and suppresses pancreatic cancer stemness, ultimately resensitizing tumors to gemcitabine. These results highlight the critical role of autophagy in maintaining cancer stemness‐driven chemoresistance in PDAC. While developmental pathways such as Notch, Hedgehog, and Wnt/β‐catenin are established stemness regulators [61, 62], the mechanistic interface between autophagy and stemness maintenance remains incompletely resolved. Our previous work revealed that inhibition of autophagy attenuated STYK1‐induced activation of the Wnt/β‐catenin pathway [46], suggesting that STYK1 promotes pancreatic cancer stemness by activating autophagy, which in turn potentiates Wnt/β‐catenin signaling. Collectively, we extend this concept by showing that STYK1‐dependent autophagy is functionally required for stemness maintenance and gemcitabine resistance. These findings provide a mechanistic link among STYK1, autophagy, and pancreatic cancer stemness, and suggest that targeting this regulatory network may be an effective strategy for overcoming gemcitabine resistance.

Mechanistically, our study reveals a unique signaling axis linking STYK1, AMPK, and autophagy to pancreatic cancer stemness. AMPK is a central energy sensor that maintains metabolic homeostasis under nutrient deprivation, hypoxia, and therapeutic stress, and critically orchestrates pancreatic cancer progression and therapeutic resistance. Compelling evidence indicates that AMPK activation sustains cancer stemness through multiple complementary mechanisms [63, 64]. First, AMPK promotes metabolic flexibility by enhancing glycolysis, mitochondrial quality control, and fatty acid oxidation, thereby sustaining energy production to meet the metabolic demands of stemness‐maintaining populations under nutrient‐deprived and hypoxic conditions [65]. Second, AMPK promotes autophagic flux by activating ULK1 and facilitating autophagosome biogenesis, which enables the recycling of damaged organelles and proteins to support stemness resilience during therapeutic stress [66]. In addition, AMPK phosphorylates key transcriptional regulators such as FOXO3 and NRF2, which in turn upregulate core stemness genes including *OCT4*, *SOX2*, and *NANOG* [67, 68]. Moreover, AMPK‐driven autophagy has been shown to reinforce stemness quiescence and drug resistance by suppressing mTORC1 signaling and upregulating cell cycle inhibitors such as p27 [66]. Consistent with these findings, our data support a model in which gemcitabine‐induced AMPK activation promotes STYK1 phosphorylation at Ser91 and Ser304, leading to enhanced STYK1‐mediated autophagic flux and stemness maintenance, which facilitate the development of chemoresistance in pancreatic cancer.

Current therapeutic strategies for PDAC often rely on combinatorial regimens to enhance the clinical efficacy of gemcitabine, including co‐treatment with paclitaxel, autophagy inhibitors such as chloroquine [69], or molecularly targeted agents [70]. However, these approaches are frequently limited by compensatory signaling rewiring and dose‐limiting toxicities. In this context, peptide‐based therapeutics have gained attention due to their improved specificity and favorable safety profiles. For example, SAH‐mAH2‐5, a stapled peptide targeting the microprotein N1ICD, was shown to synergize with gemcitabine, significantly reducing tumor burden and prolonging survival in preclinical PDAC models [71]. Similarly, our rationally designed STYK1‐targeting peptide inhibitor SAIDP5 delays the onset of gemcitabine resistance and suppresses PDAC progression when combined with gemcitabine. Interestingly, we found that SAIDP5 shares the same amino acid sequence as SkP5, a STYK1‐derived peptide previously shown to inhibit the Wnt/β‐catenin signaling pathway [46]. This dual functionality reinforces the therapeutic potential of SAIDP5 in concurrently targeting both autophagy and Wnt/β‐catenin pathways to suppress PDAC growth. This dual functionality suggests that SAIDP5 may exert antitumor activity by simultaneously interfering with STYK1‐dependent autophagy and Wnt/β‐catenin signaling. Such a property may be advantageous in PDAC, where chemoresistance is rarely driven by a single pathway but instead emerges from coordinated changes in survival signaling, stemness plasticity, autophagy, and tumor–microenvironment interactions. Nevertheless, the precise mechanism by which STYK1 affects gemcitabine resistance in pancreatic cancer through crosstalk between autophagy and Wnt/β‐catenin signaling pathways will be further defined in future studies. Although SAIDP5 exhibits considerable promise as a therapeutic candidate, challenges related to peptide stability in plasma, immunogenicity, delivery efficiency, and target specificity remain to be addressed for clinical translation. In addition, although SAIDP5 was designed to disrupt the STYK1–AMPK interaction and exhibited clear gemcitabine‐sensitizing activity in PDAC models, short peptides may interact with unintended proteins that share similar binding motifs, charge distributions, or structural features [72, 73]. Therefore, potential off‐target effects cannot be completely excluded at this stage. Future studies should incorporate scrambled peptide controls, binding‐deficient peptide mutants, peptide pull‐down followed by mass spectrometry, and global proteomic or phosphoproteomic analysis to define the target spectrum and specificity of SAIDP5 [72, 74]. Moreover, evaluating SAIDP5 in normal pancreatic epithelial cells, patient‐derived organoids, PDX models, and major non‐tumor tissues will be essential for determining its therapeutic selectivity and safety window [72, 73, 75]. Taken together, our findings establish STYK1 as a critical mediator of gemcitabine resistance in PDAC and propose SAIDP5‐based combination therapy may represent a mechanistically informed strategy to improve gemcitabine responsiveness by attenuating autophagy‐ and stemness‐associated chemoresistance.

## 4 Materials and Methods

### 4.1 Establishment of Subcutaneous Xenograft Model

For the subcutaneous xenograft model, 3 × 10^6^ PANC‐1/GEM or AsPC‐1/GEM cells stably STYK1 knockdown or PANC‐1/GEM or AsPC‐1/GEM cells were injected subcutaneously into the bilateral axillary regions of 4‐week‐old female BALB/c nude mice, with five mice in each group.

### 4.2 Establishment of Gemcitabine‐Resistant PDX

To establish a gemcitabine‐resistant patient‐derived xenograft (PDX) model, fresh tumor specimens from patients with pathologically confirmed pancreatic cancer were aseptically cut into approximately 3 mm^3^ fragments and subcutaneously implanted into the axillary region of 8‐week‐old female BALB/c nude mice. When tumors reached approximately 1 cm^3^, first‐generation tumors (P1) were harvested and passaged to generate P2 tumors, followed by serial passaging to P5. During passages P3–P5, mice bearing tumors of approximately 4 × 4 × 4 mm^3^ received gemcitabine (50 mg/kg, intraperitoneally, twice weekly) or PBS. After three consecutive generations of gemcitabine treatment, the tumors developed resistance to gemcitabine. Therefore, gemcitabine‐treated P5 tumors were designated as gemcitabine‐resistant PDX models. This model was provided by the Laboratory Animal Center of Sun Yat‐sen University [76].

### 4.3 Drug Treatment Experiment

When xenograft or PDX tumors reached approximately 4 × 4 × 4 mm^3^, the animals were treated with 10% DMSO, gemcitabine (25 mg/kg body weight), commercially synthetic peptides (25 mg/kg body weight), or combination therapy via intraperitoneal injection twice a week. Tumor volumes were monitored every 5 days or 10 days via caliper measurements and calculated as V = [(length × width ^ 2)/2]. Endpoint procedures were initiated when tumors reached 1,500 mm^3^, with subsequent tumor excision and weight recording for downstream analysis.

### 4.4 KPC Mouse Model Experiments

The establishment of classical *LSL‐Kras^G12D/+^*; *LSL‐Trp53^R172H/+^*; *Pdx1^Cre^*; *Styk1^−/−^* mice (KPCS mice) is as previously described [46]. Later, two‐month‐old KPC mice with similar body weights were selected and randomly divided into four groups, which were treated with gemcitabine (25 mg/kg body weight, twice a week), CP‐SAIDP5 peptide (25 mg/kg body weight, twice a week), or combination therapy for four weeks. After the treatment period, tumor tissues and heart blood were harvested for indicator analysis.

### 4.5 Establishment of Gemcitabine‐Resistant Cell Lines

Gemcitabine‐resistant PANC‐1/GEM and AsPC‐1/GEM cell lines were established by stepwise drug‐gradient induction. Briefly, PANC‐1 and AsPC‐1 cells in the logarithmic growth phase were first used to determine the half‐maximal inhibitory concentration (IC50) of gemcitabine by CCK‐8 assay. The corresponding IC50 value was used as the initial induction concentration. Cells were exposed to gemcitabine‐containing complete medium for 24 h, after which the medium was removed. Cells were washed twice with PBS and cultured in fresh drug‐free complete medium. The next round of induction was performed only after the cells had recovered stable morphology and proliferation. The gemcitabine concentration was then gradually increased in twofold increments. After approximately 10 months of continuous selection, cells capable of stable growth for 4 days in complete medium containing a high concentration of gemcitabine were considered gemcitabine‐resistant and designated PANC‐1/GEM and AsPC‐1/GEM cells. Resistance was further confirmed by determining the IC50 values of the resistant cell lines using the CCK‐8 assay.

### 4.6 Immunohistochemical (IHC)

Pancreatic tumor tissues were sectioned at a thickness of 3.5 µm and performed with Hematoxylin and eosin (H&E) staining according to the manufacturer's instructions. Tissue sections were blocked with 5% normal goat serum with 0.1% Triton X‐100 and 3% H_2_O_2_ in PBS for 60 min at room temperature and then incubated with anti‐ki67 (GB111499‐100, Servicebio), anti‐CK19 (GB11197, Servicebio), anti‐CD44 (15675‐1‐AP, Proteintech), anti‐CD133 (18470‐1‐AP, Proteintech), anti‐LC3 (14600‐1‐AP, Proteintech), anti‐p62 (66184‐1‐Ig, Proteintech) overnight, which were then detected by HRP conjugated DAB.

### 4.7 GST Affinity‐isolation Assay and Surface Plasmon Resonance

GST affinity‐isolation assay was performed as described previously [24]. The cDNA encoding STYK1 ICD (aa 26–422) or AMPK was cloned into pGEX4T‐1 (GE, 27‐4580‐01). GST‐tagged fusion proteins were incubated with glutathione‐Sepharose beads at 4°C, followed by elution with reduced glutathione. Expression of GST fusion proteins was confirmed by a Western blotting assay. Binding kinetics between GST‐tagged AMPK and commercially synthesized α‐helical peptides were measured by surface plasmon resonance using a Biacore T200 instrument (GE Healthcare, Chicago, IL). The dissociation constant was calculated according to BIA evaluation software (GE Healthcare).

### 4.8 Tumor Sphere Formation Assays

Single‐cell suspensions of AsPC‐1/GEM cells with stable STYK1 knockdown, or AsPC‐1 cells stably overexpressing STYK1 and its mutants or AsPC‐1/GEM cells, or AsPC‐1 cells with stable ATG7 knockdown (5×10^2 cells/mL) were cultured in ultra‐low attachment 96‐well plates (Corning, Amercia) in phenol red‐free DMEM/F12 (Servicebio, G4612), supplemented with B27 Supplement (Gibico, USA, #12587010), 20 ng/mL epidermal growth factor (EGF; UA BIOSCIENCE, China, UA040276) and 20 ng/mL basic fibroblast growth factor (bFGF; UA BIOSCIENCE, China, UA040003). After 7–14 days, tumor spheroids were observed using an Olympus FSX100 microscope.

### 4.9 Flow Cytometry

PANC‐1/GEM and AsPC‐1/GEM cells with stable STYK1 knockdown, or PANC‐1 and AsPC‐1 cells stably overexpressing STYK1 and its mutants or PANC‐1/GEM and AsPC‐1/GEM cells, or PANC‐1 and AsPC‐1 cells with stable ATG7 knockdown were plated in 6‐well plates and cultured until 80% confluence. After treatment with gemcitabine, CQ, or CP‐SAIDP5 peptide for the corresponding duration, the cells were digested with 0.25% trypsin and collected at a concentration of 10^6 cells. Apoptosis was assessed using an Annexin V‐FITC Apoptosis Detection Kit (Elabscience, E‐CK‐A252). All samples were stained with Annexin V‐FITC and propidium iodide (PI), then filtered through a mesh to remove aggregates prior to analysis. For cell surface marker staining, 100 µL of diluted anti‐CD44‐FITC antibody (560977, BD Pharmingen) and anti‐CD133‐APC antibody (566597, BD Pharmingen) in staining buffer were added to the cell suspension, followed by incubation at 4 °C for 20 min in the dark. After washing with PBS, cells were analyzed by flow cytometry.

### 4.10 Statistical Analysis

All experiments were conducted at least three times independently. Statistical analyses were performed using GraphPad Prism 6.0 software (GraphPad, La Jolla, CA, USA). Data are presented as the mean ± SD (standard deviation) of three independent replicates. A *p*‐value of < 0.05 was considered statistically significant. Differences between two groups were assessed using an independent sample *t*‐test (two‐tailed), while differences among multiple groups were analyzed using one‐way ANOVA. Correlations between variables were assessed using Spearman's correlation coefficient. For all statistical analyses, * represents *p* < 0.05, ** represents *p* < 0.01, *** represents *p* < 0.001, and **** represents *p* < 0.0001.

## Author Contributions

X.Y.D., S.L., and C.S.H. wrote the manuscript and performed molecular biology experiments. J.F.T., C.F.Z. and Y.Z. designed the whole project and supervised all experiments. X.Y.D., S.L., C.S.H., X.H.Q., J.Y.G., G.F.W., Q.Z., H.F.T., Y.H. R.Z., H.L., S.X., D.G., C.J.Y., W.Y.Q., and C.C.L. conducted all experiments and analyzed the data. X.Z.C. provided support with experimental and clinical techniques. All authors read and approved the final manuscript.

## Ethics Statement

All animal procedures were conducted in compliance with institutional guidelines and approved by the Animal Care and Use Committee of Hubei University of Technology (NO: HBUTXM20250327).

## Conflicts of Interest

The authors declare no conflicts of interest.

## Supporting information




**Supporting File**: advs78152‐sup‐0001‐SuppMat.docx.

## Data Availability

The data supporting this study are available within the paper and its Supplementary Data file. The RNA sequencing data in this study have been deposited in NCBI's Gene Expression Omnibus (GEO) under accession code GSE348513.
