# Supplementary material for: Phosphorylated STYK1 Promotes Gemcitabine Resistance via Autophagy‐Mediated Cancer Stemness in Pancreatic Cancer
Source: Adv Sci (Weinh). 2026 Oct 11:e21488. Online ahead of print. doi: 10.1002/advs.202521488 (PMC13655253; doi:10.1002/advs.202521488)
Supplement: Supplementary file 1 [file ADVS-9999-e21488-s001.docx]

Supplementary Materials for

Phosphorylated STYK1 Promotes Gemcitabine Resistance via Autophagy-Mediated Cancer Stemness in Pancreatic Cancer

Xueying Dong^1,2#^, Shi Li^1#^, Chensong Huang^3#^, Xuehong Qian^4^, Jieyu Guo^1^, Guifang Wu^1^, Qi Zhang^1^, Hongfei Tan^1^, Yu Han^2^, Rui Zhang^1^, Hao Lyu^1^, Shuai Xiao^1^, Dong Guo^1^, Chaojun Yan^1^, Wenying Qin^1^, Changchun Li^2^, Xing-Zhen Chen^5^, Ying Zhao^6*^, Cefan Zhou^1,5*^ and Jingfeng Tang^1, 5*^

Correspondence to: [Jingfeng_hut@163.com](mailto:Jingfeng_hut@163.com); [cefan@hbut.edu.cn](mailto:cefan@hbut.edu.cn); zhaoying0812@bjmu.edu.cn

**This PDF file includes:**

Materials and Methods

Figures. S1 to S8

**Materials and methods**

**Cell Lines and Reagents**

Human pancreatic cancer cell lines PANC-1 (RRID: CVCL_0480, 2022-09-06), HEK293T (RRID: CVCL_0063, 2022-04-14), AsPC-1 (RRID: CVCL_0152, 2022-09-06) and HeLa (CVCL_0030, 2022-05-15) cells were purchased from the cell center of the Institute of Biochemistry and Cell Biology, Chinese Academy of Sciences (Shanghai, China), the gemcitabine-resistant PANC-1/GEM (RRID: CVCL_C0WS, 2022-09-06) and AsPC-1/GEM cell lines were derived from parental PANC-1 cells. All cell lines were confirmed contamination-free by mycoplasma PCR testing and STR profiling. Cells were cultured in Dulbecco’s Modified Eagle’s Medium (DMEM) (Servicebio, G4511) supplemented with 10% fetal bovine serum (Gibco, USA), 100 U/ml penicillin G, and 100 μg/ml streptomycin (Biosharp, BL505A) at 37°C in a humidified incubator with 5% CO2. The autophagy inhibitor chloroquine diphosphate salt (CQ) was purchased from Sangon Biotech (A506569). Gemcitabine, the AMPK activator AICAR, and the AMPK inhibitor Compound C were purchased from MedChemExpress (USA, LY 188011, HY-13417, HY-13418A). CP-SAIDP5 was synthesized by GenScript. The starvation treatment medium Earle's Balanced Salt Solution (EBSS) was purchased from Servicebio (G4214).

**Plasmids, shRNAs and Transfection**

DNA fragments encoding STYK1, AMPK, BECN1, ATG14, ZFYVE1 and WIPI1 amplified by PCR were gifts from Prof. Jiahuai Han (Xiamen University, China). The PCR products of STYK1 were cloned into pcDNA3.1-3×HA, pCMV-3×Flag (Sigma, E4401), and pCMV-mCherry (constructed by our lab). The PCR products of AMPK were cloned into pcDNA3.1-3×HA and pCMV-N1-GFP (Clontech, 60851). The PCR products of Beclin1 and ATG14L were cloned into pCMV-3×Flag. The PCR products of ZFYVE1 and WIPI1 were cloned into pCMV-N1-GFP. ptfLC3 (mammalian expression of rat LC3 fused to mRFP and GFP) was a gift from Prof. Tamotsu Yoshimori (Addgene, 21074). To make STYK1 mutants or truncations, a series of forwarding primers harboring the desired mutations were used in PCRs. The nucleotide sequences of all constructs were confirmed by DNA sequencing. DNA and interference RNA were transfected with Lipofectamine 3000 (Invitrogen, L3000015) according to the manufacturer’s instructions. Knockdown targets of STYK1#1: 5ʹ- AGCTGGGAAGCAGGACATGGAGG -3ʹ; STYK1#2: 5ʹ- GCCACTTAAGGAGACATCCG -3ʹ; STYK1#3: 5ʹ- GCCACAGGATGACCCCAAGA -3ʹ. Knockdown targets of ATG7: 5ʹ- AGAAGAAGCTGAACGAGTAT -3ʹ. shRNA for AMPK was 5ʹ- GAAGCAGTATGAAGTGATTATCTCGAGATAATCACTTCATACTGCTTC -3ʹ.

**Western Blotting**

Whole-cell extracts were prepared with 1% sodium dodecyl sulfate (SDS) lysis buffer with freshly added 1% proteinase inhibitor cocktail (Biomake, B14001) and 1mM phenylmethylsulfonyl fluoride (PMSF, MCE, HY-B0496). Western blotting was performed as described previously^77^. The following primary antibodies were used: anti-CD44 (15675-1-AP, Proteintech), anti-CD133 (18470-1-AP, Proteintech), anti-ATG7 (10088-2-AP, Proteintech), anti-GAPDH (60004-1-Ig, Proteintech), anti-AMPK (18167-1-AP, Proteintech), anti-VPS34 (13723-1-AP, Proteintech), anti-VPS15 (17894-1-AP, Proteintech), anti-Beclin1 (11306-1-AP, Proteintech), anti-ATG14L (28021-1-AP, Proteintech), anti-mouse HA (M180-3, MBL), anti-Rabbit HA (51064-2-AP, Proteintech), anti-mouse DYKDDDDK (M185, MBL), anti-Rabbit DYKDDDDK (80010-1-RR, Proteintech), anti-MYC (60003-2-Ig, Proteintech), anti-ULK1 (20986-1-AP, Proteintech), anti-GST (66001-2-Ig, Proteintech), anti-GFP (50430-2-AP, Proteintech), anti-p62 (66184-1-Ig, Proteintech), anti-phospho-AMPKα (Thr172) (2535T, Cell Signaling Technology), anti-phospho-ULK1 (Ser317) (89267T, Cell Signaling Technology), anti-STYK1 (862318, zenbio), anti-LC3B (381544, zenbio), anti-phosphoserine (AB1603, Sigma-Aldrich).

**Co-immunoprecipitation (Co-IP)**

Co-IP analysis was performed as previously described [1]. Briefly, cells were harvested and lysed in RIPA buffer (50 mM Tris-HCl, pH 7.5, 150 mM NaCl, 1% NP40 [Applygen, B1013], and 2 mM EDTA), supplemented with proteinase inhibitor cocktail (Selleck, B14001) and Halt phosphatase inhibitor cocktail (Thermo Fisher Scientific, 78420). The supernatants of the cell lysates were pre-cleared with protein A/G Magic Beads (Selleck, B23202) and then incubated overnight with primary antibodies, as per the experimental design. The mixtures were subsequently incubated with protein A/G Magic Beads for 2 hours at 4 °C. The immunoprecipitate was then analyzed by western blotting.

**Immunofluorescence**

PANC-1, PANC-1/GEM, or HeLa cells were fixed with 4% formaldehyde for 10-15 min, then permeabilized with 0.2% Triton X-100 in PBS for 15 min. The cells were subsequently blocked with 3% BSA in PBS for 45-60 min at room temperature. Immunostaining was performed using the specified antibodies, and the nuclei were counterstained with DAPI. Images were captured using a Leica inverted confocal SP8 microscope (Leica Microsystems, Germany).

**Alcian Blue and Sirius Red Staining**

Pancreatic tissue sections were deparaffinized in xylene and rehydrated through a graded ethanol series. For acidic mucopolysaccharide detection, sections were incubated with alcian blue solution (Vector Laboratories, H-3501) for 30 min at room temperature. Following thorough rinsing with distilled water, counterstaining was performed using either nuclear fast red or eosin. Strongly acidic mucosubstances were identified by distinct blue staining. Collagen fibers were subsequently stained with Picro-Sirius red solution (Sigma, S365548). After staining, sections were rinsed twice in acidified water (pH 2.0 adjusted with 0.5% acetic acid), dehydrated with 100% ethanol, cleared in xylene, and mounted with resinous medium.

**Hematoxylin and eosin (H&E) staining**

Pancreatic tissues or subcutaneous tumor sections were deparaffinized and rehydrated, followed by hematoxylin staining for nuclear visualization and eosin staining for cytoplasm and extracellular matrix visualization. After staining, sections were examined using a light microscope, and representative images were captured.

**RNA Extraction and qRT-PCR**

Total RNA was extracted from cultured pancreatic cancer cells using Trizol reagent (Invitrogen) according to the manufacturer’s protocol. qRT-PCR was performed as previously described [2]. The *mRNA* expression level for each sample was normalized to the expression of GAPDH using the 2 ^-ΔΔct^ method [3] with three biological replicates of comparative qRT-PCR. The following primer sequences were used for qRT-PCR: GAPDH, (forward) 5′- GGGGAGCCAAAAGGGTCATCATCT -3′ and (reverse) 5′- GACGCCTGCTTCACCACCTTCTTG -3′; STYK1, (forward) 5′- TCGAGCCAATATGAACACTGGG -3′ and (reverse) 5′- TCGCCCTAAGAAATCTTGTACCT -3′; SOX2, (forward) 5′- TGGACAGTTACGCGCACAT -3′ and (reverse) 5′- CGAGTAGGACATGCTGTAGGT -3′; CD133, (forward) 5′- AGTCGGAAACTGGCAGATAGC -3′ and (reverse) 5′- GGTAGTGTTGTACTGGGCCAAT -3′; CD44, (forward) 5′- CCAGAAGGAACAGTGGTTTGGC -3′ and (reverse) 5′- ACTGTCCTCTGGGCTTGGTGTT -3′; NANOG, (forward) 5′- CTCCAACATCCTGAACCTCAGC -3′ and (reverse) 5′- CGTCACACCATTGCTATTCTTCG -3′; OCT4, (forward) 5′- CCTGAAGCAGAAGAGGATCACC -3′ and (reverse) 5′- AAAGCGGCAGATGGTCGTTTGG -3′. Amplification was performed in an ABI Q3 real-time PCR system using a SYBR Green master mix (YEASEN, 11201ES08).

**Lentiviral Production and Creation of Stable Cell Lines**

Knockdown STYK1 and AMPK shRNA were subcloned into the lentiviral vector lentiCRISPR v2 and pLKO.1-puro, and Flag-tagged STYK1 and its mutants were subcloned into the lentiviral vector pCDH-CMV-MCS-EF1-turboRFP-T2A-Puro or pCDH-CMV-MCS-EF1-Puro, which were co-transfected with viral packaging plasmids psPAX2 and pMD2.G into 293T cells in 10 cm dishes. After 48 h of transfection, the viral supernatant was collected and filtered through a 0.22 µm membrane. After applying the viral supernatant to PANC-1, AsPC-1, PANC-1/GEM or AsPC-1/GEM cells with 10 μg/μl of polybrene (Solarbio, H8761), selection for puromycin (Solarbio, IP1280) resistance was initiated 48 h after infection. The selection medium was changed every 1-2 days for several weeks, and clones of puromycin-resistant cells were isolated and expanded for further characterization.

**5-Ethynyl-20-deoxyuridine (EdU) Incorporation Assay**

EdU-labeled PANC-1 and AsPC-1 cells stably overexpressing STYK1 and its mutants, or PANC-1/GEM and AsPC-1/GEM cells with stable STYK1 knockdown, or PANC-1/GEM and AsPC-1/GEM cells treated with gemcitabine and/or CP-SAIDP5 peptide. Cell proliferation was assessed using the BeyoClick™ EdU Cell Proliferation Kit and Alexa Fluor 555 or 488 (Beyotime, C0075S, C0071S). Cells were photographed under an Olympus FSX100 microscope.

**Wound-healing Assay**

PANC-1 and AsPC-1 cells stably overexpressing STYK1 and its mutants, or PANC-1/GEM and AsPC-1/GEM cells with stable STYK1 knockdown, or PANC-1/GEM and AsPC-1/GEM cells were placed in 6 - well plates and cultured to 90% confluence. A straight wound through the cell layer was made with a sterile pipette tip. Then, Cells were treated with gemcitabine or CP-SAIDP5 peptide. Images were captured using an Olympus FSX100 microscope. Cell migration was analyzed by measuring the wound closure rate.

**Trans-well Assays**

PANC-1 and AsPC-1 cells stably overexpressing STYK1 and its mutants, or PANC-1/GEM and AsPC-1/GEM cells with stable STYK1 knockdown, or PANC-1/GEM and AsPC-1/GEM cells were placed in the upper well of an uncoated trans-well chamber with an 8-µm φ pore membrane. DMEM culture medium containing 20% FBS and treated with gemcitabine or CP-SAIDP5 peptide were then added to the lower chamber of the trans-well chamber. Before the invasion assay, 10 mL of Matrigel® (Corning, USA, #356234) was thawed overnight at 4 °C. The matrix was diluted with serum-free growth medium at a ratio of 1:9. 100μL the chilled diluted Matrigel was placed in the upper chamber of the Trans-well. The culture dish was then placed in a 37 °C incubator for 60 min to allow it to solidify. Cells were then seeded as described above. The plates were incubated at 37 °C for 24 h. After the removal of all non-migrated or non-invasive cells, the migrated or invasive cells were stained using crystal violet. A microscope was employed to capture the images and count the migrated cells.

**Colony Formation Assay**

Colony formation assay was performed as described previously [2]. PANC-1 and AsPC-1 cells stably overexpressing STYK1 and its mutants were incubated with a complete medium at 37 °C for 2-3 weeks. The cells were fixed with 4% paraformaldehyde and stained with 2% crystal violet. The images were obtained and the number of colonies was counted. Each experiment was repeated in triplicate.

**CCK-8 Assay**

Cell viability was measured using the CCK-8 assay kit (MedChemExpress, America, #HY-K0301). A total of 2000 cells were seeded per well in a 96-well plate. After incubation for 0, 24, 48, and 72 hours, the culture medium was removed and replaced with fresh medium containing CCK-8 reagent for 2 hours. Absorbance was then measured at 450 nm using a microplate reader (BioTek, America).

**RNA-sequencing**

Total RNA was extracted from control and stable STYK1 knockdown AsPC-1/GEM cells using Trizol reagent, with three biological replicates included in each group. The mRNA was enriched from total RNA using Oligo(dT) magnetic beads and then randomly fragmented in Fragmentation Buffer in the presence of divalent cations. After fragmentation, first-strand cDNA was synthesized using random hexamer primers. Following end repair, A-tailing, adaptor ligation, fragment size selection, amplification, and purification, the libraries were sequenced on the NovaSeq X Plus platform. The raw data has been uploaded to NCBI’s Gene Expression Omnibus (GEO) under accession code GSE348513.

**Synthesis of SAIDP5 peptide**

SAIDP5 peptide (YGRKKRRQRRRI-RADVWSFGILLYEMV) was commercially synthesized by GenScript (Nanjing, China) with a purity of >95%. The peptide contains an N-terminal TAT cell-penetrating sequence (YGRKKRRQRRRI) fused to the functional peptide sequence RADVWSFGILLYEMV. The N-terminus was acetylated and the C-terminus was amidated. The peptide was dissolved in sterile water and stored at -20 °C until use.

 **Fig S1. STYK1 promotes gemcitabine resistance in pancreatic cancer cell, related with Fig.1. A)** Heatmap of *STYK1* expression levels in MIAPACA2 and PANC-1 cells exposed to gemcitabine at varying concentrations and time. **B, C)** RT-qPCR analysis of *STYK1 mRNA* levels in AsPC-1 cells treated with gemcitabine at different concentrations and time courses. ****P < 0.0001 according to Student's *t*-test. **D, E)** Cell viability of PANC-1 and PANC-1/GEM cells, as well as AsPC-1 and AsPC-1/GEM cells, were treated with different concentrations of gemcitabine for 72 h, as assessed by CCK-8 assay. **F, G)** Western blotting analysis confirmed the efficiency of STYK1 knockdown in PANC-1/GEM and AsPC-1/GEM cells and STYK1 overexpression in PANC-1 and AsPC-1 cells. **H, I)** AsPC-1/GEM cells with stable STYK1 knockdown and AsPC-1 cells stably overexpressing STYK1 were treated with gemcitabine (40 μM for H or 1 μM for I) for different durations as indicated, and OD values was assessed using the CCK-8 assay. ****P < 0.0001 according to Student's *t*-test. **J-L)** Apoptosis analysis of PANC-1 and AsPC-1 cells stably overexpressing STYK1 following treatment with or without gemcitabine (1 μM, 24 h), assessed by an Annexin V-AbFluor™ 488/PI double staining Apoptosis Detection Kit. Annexin V-positive cells indicate early apoptosis, whereas PI-positive cells indicate late apoptosis/necrosis (n = 3).  *P < 0.05; **P < 0.01; ***P < 0.001 according to Student's *t*-test. **M-P)** Representative images and quantitative analysis of DNA synthesis in PANC-1/GEM cells with stable STYK1 knockdown or PANC-1 cells stably overexpressing STYK1 treated with or without gemcitabine (5 μΜ for M, or 1 μΜ for O, 24 h) (n = 3). Scale bar: 100 μm. **P < 0.01; ***P < 0.001; according to Student's *t*-test. **Q-X)** Representative images and quantitative analysis of Trans-well assays with and without matrigel of PANC-1/GEM and AsPC-1/GEM cells with stable STYK1 knockdown, as well as PANC-1 and AsPC-1 cells stably overexpressing STYK1, following treatment with or without gemcitabine (5 μM for Q, 40 μΜ for S, or 1 μΜ for U and W, 24 h) (n = 3). Scale bar: 100 μm. *P < 0.05; **P < 0.01; ***P < 0.001; ****P < 0.0001 according to Student's *t*-test. Data were expressed as mean ± SD.

 **Fig S2. STYK1 facilitates pancreatic cancer stemness upon gemcitabine treatment by promoting autophagy activation, related with Fig.2. A)** Correlation analysis using GEPIA2 revealed that STYK1 expression was positively correlated with CD44 (R=0.22, P=0.0028) and CD133 (R=0.27, P<0.001) in pancreatic cancer. **(B)** Western blotting analysis of CD44, CD133 and LC3 protein expression levels in PANC-1 and AsPC-1 cells with stable STYK1 overexpression treated with or without gemcitabine (1 μM, 24 h). **C, D)** RT-qPCR analysis of *CD44*, *CD133*, and stemness-associated transcription factors *OCT4*, *NANOG*, and *SOX2* in PANC-1 and AsPC-1 cells with stable STYK1 overexpression treated with or without gemcitabine (1 μM, 24 h) (n = 3). *P < 0.05; **P < 0.01; ***P < 0.001; ****P < 0.0001 according to Student's *t*-test. **E)** Representative images of AsPC-1 cells with stable STYK1 overexpression after 14 days of culture in serum-free medium treated with or without gemcitabine (1 μM). The number of spheroids with a diameter of >70μm was statistically analyzed (n = 3). Scale bar: 100 μm. *P < 0.05; ***P < 0.001 according to Student's *t*-test. **F)** Flow cytometric analysis of CD44^+^CD133^+^ cells in PANC-1 and AsPC-1 cells with stable STYK1 overexpression treated with or without gemcitabine (1 μM, 24 h). Numbers in the graphs indicate the percentage of the subpopulation (n = 3). **G)** Western blotting analysis of CD44, CD133 and LC3 protein expression levels in PANC-1/GEM and AsPC-1/GEM cells with stable STYK1 knockdown treated with or without autophagy inhibitor CQ (10 μM, 6 h). **H, I)** RT-qPCR analysis of *CD44*, *CD133*, and *OCT4*, *NANOG*, *SOX2* in PANC-1/GEM and AsPC-1/GEM cells with stable STYK1 knockdown treated with or without autophagy inhibitor CQ (10 μM, 6 h) (n = 3). **L)** Quantification of spheroids with a diameter greater than 70 μm formed by PANC-1/GEM and AsPC-1/GEM cells with stable STYK1 knockdown treated with or without autophagy inhibitor CQ (10 μM, 6 h) (n = 3). *P < 0.05; **P < 0.01; ***P < 0.001; ns (no significance) according to Student's *t*-test. **J)** Flow cytometric analysis of CD44^+^CD133^+^ cells in PANC-1/GEM and AsPC-1/GEM cells with stable STYK1 knockdown treated with or without autophagy inhibitor CQ (10 μM, 6 h). Numbers in the graphs indicate the percentage of the subpopulation (n = 3). Data were expressed as mean ± SD, *P < 0.05; **P < 0.01; ***P < 0.001; ****P < 0.0001; ns (no significance) according to Student's *t*-test.

 **Fig S3. Gemcitabine induced AMPK to amplify STYK1-mediated autophagy activation, related with Fig.3. A-D)** Western blotting analysis and quantification of LC3, p62, STYK1, and p-AMPK T172 protein expression levels in PANC-1 cells with wildtype and stably overexpressing STYK1 treated with gemcitabine at different concentrations and for different durations (n = 3). *P < 0.05; **P < 0.01; ***P < 0.001; ****P < 0.0001 according to Student's *t*-test. **E-H)** Western blotting analysis and quantification of LC3 and p-AMPK T172 protein expression levels in PANC-1/GEM cells with wildtype and stable STYK1 knockdown treated with or without the EBSS (2 h) and AMPK inhibitor compound C (10 μM, 24 h) (E) or the AMPK activator AICAR (1 mM, 4 h) (G) (n = 3). *P < 0.05; ***P < 0.001; ****P < 0.0001 according to Student's *t*-test. **(I)** Co-immunoprecipitation analysis of the interaction between ATG14L and VPS34, VPS15, and Beclin1 in PANC-1/GEM cells with stable STYK1 knockdown, treated with or without gemcitabine (5 μM, 24 h). Cell lysates were used for IP and Western blotting with the indicated antibodies. Data were expressed as mean ± SD, *P < 0.05; **P < 0.01; ***P < 0.001; ****P < 0.0001 according to Student's *t*-test.

 **Fig S4. AMPK directly binds and phosphorylates STYK1 at S91 and S304, related with Fig.4. A, B)** Co-immunoprecipitation analysis of the interaction between STYK1 and AMPK in HEK293T cells treated with or without the AMPK activator AICAR (1 mM, 4 h). Cell lysates were used for IP and Western blotting with the indicated antibodies. **C-F)** Co-immunoprecipitation analysis of the interaction between STYK1 and AMPK or its total serine phosphorylation level of STYK1 in PANC-1 cells subjected to time-dependent EBSS starvation. Cell lysates were used for IP and Western blotting with the indicated antibodies. Co-immunoprecipitated AMPK levels and total serine phosphorylation level were quantified and normalized to immunoprecipitated STYK1 levels (n = 3). *P < 0.05; ***P < 0.001; ****P < 0.0001 according to Student's *t*-test. **G, H)** The total serine phosphorylation levels of STYK1 and its mutants were measured in HEK293T cells co-transfected with Flag-tagged wild-type or mutant STYK1 constructs and AMPK depletion (G), or HA-tagged AMPK or constitutively active HA-AMPK-CA (H). Cell lysates were used for IP and Western blotting with the indicated antibodies. **(I)** Total serine phosphorylation levels of purified GST-STYK1-ICD and its mutants were incubated with HA-AMPK-CA (T172D) obtained from HEK293T cell lysates. Data were expressed as mean ± SD, *P < 0.05; ***P < 0.001; ****P < 0.0001 according to Student's *t*-test.

**Fig S5. Phosphorylated STYK1 future promote autophagy, cancer stemness and pancreatic cancer progression, related with Fig.5. A, B)** Western blotting and quantitative analysis of LC3 and p62 expression in PANC-1 cells stably overexpressing STYK1 and its mutants treated with or without gemcitabine (1 μM, 24 h) or autophagy inhibitor CQ (10 μM, 6 h) (n = 3). *P < 0.05; **P < 0.01; ***P < 0.001; ****P < 0.0001 according to Student's *t*-test. **C, D)** Representative confocal images and quantification of GFP-ZFYVE1 or GFP-WIPI1 puncta in PANC-1 cells overexpressing Flag-STYK1 and its mutants. The number of their spots was quantified (n = 8). Scale bar: 10 μm. *P < 0.05; **P < 0.01; ***P < 0.001; ****P < 0.0001 according to Student's *t*-test. **E)** Co-immunoprecipitation analysis of the interactions between Beclin1, VPS34 and STYK1 after overexpression Flag-STYK1 and its mutants in HEK293T cells. Cell lysates were used for IP and Western blotting with the indicated antibodies. **F)** RT-qPCR analysis of *CD44*, *CD133*, and stemness-associated transcription factors *OCT4*, *NANOG*, and *SOX2* in AsPC-1 cells stably overexpressing STYK1 and its mutants (n = 3). *P < 0.05; **P < 0.01; ***P < 0.001; ****P < 0.0001 according to Student's *t*-test. **G, H)** Flow cytometric analysis and quantification of CD44^+^CD133^+^ cells in AsPC-1 cells stably overexpressing STYK1 and its mutants. Numbers in the graphs indicate the percentage of the subpopulation (n = 3). **P < 0.01; ***P < 0.001; ****P < 0.0001 according to Student's *t*-test. **I-L)** Representative images and quantitative analysis of Trans-well assays with and without matrigel of PANC-1 and AsPC-1 cells stably overexpressing STYK1 and its mutants (n = 3). Scale bar: 100 μm. **P < 0.01; ***P < 0.001; ****P < 0.0001 according to Student's *t*-test. **M-P)** Representative images and quantification of wound healing assays in PANC-1 and AsPC-1 cells stably overexpressing STYK1 and its mutants (n = 3). Scale bar: 200 μm. *P < 0.05; **P < 0.01; ***P < 0.001; ****P < 0.0001 according to Student's *t*-test. **Q, R)** Representative images and quantification of plate colony formation assay in PANC-1 and AsPC-1 cells stably overexpressing STYK1 and its mutants (n = 3). *P < 0.05; **P < 0.01; ***P < 0.001; ****P < 0.0001 according to Student's *t*-test. **S-V)** Representative images and quantitative analysis of DNA synthesis in PANC-1 and AsPC-1 cells stably overexpressing STYK1 and its mutants (n = 3). Scale bar: 100 μm. *P < 0.05; **P < 0.01 according to Student's *t*-test. Data were expressed as mean ± SD.

**Fig S6. Peptide disrupting STYK1-AMPK interaction inhibits autophagy and pancreatic cancer stemness, related with Fig.6. A)** Cell-penetrating SAIDP5 (CP-SAIDP5) was generated by linkage to the cell-penetrating peptide TAT, and CP-FITC-SAIDP5 was synthesized by FITC modification at its N-terminal end, and its membrane-penetrating effect was verified by confocal microscopy. Scale bar: 10 μm. **B, C)** Cell viability of PANC-1/GEM and AsPC-1/GEM cells were treated with different concentrations of gemcitabine for 72 h in the presence or absence of CP-SAIDP5, as assessed by CCK-8 assay. **D, E)** Representative confocal images and quantification of GFP-ZFYVE1 or GFP-WIPI1 puncta in PANC-1/GEM cells after treatment with peptide CP-SAIDP5 (5 μM, 24 h) or gemcitabine (5 μM, 24 h) alone or in combination. The number of spots was quantified (n = 3). Scale bar: 10 μm. **P < 0.01; ***P < 0.001; ****P < 0.0001 according to Student's *t*-test. **F)** Western blotting of CD44 and CD133 expression levels after treatment of AsPC-1/GEM cells with peptide CP-SAIDP5 (5 μM, 24 h) or gemcitabine (40 μM, 24 h) alone or in combination. **G)** RT-qPCR analysis of *CD44*, *CD133*, and stemness-associated transcription factors *OCT4*, *NANOG*, and *SOX2* after treatment of AsPC-1/GEM cells with peptide CP-SAIDP5 (5 μM, 24 h) or gemcitabine (40 μM, 24 h) alone or in combination (n = 4). **P < 0.01; ***P < 0.001; ****P < 0.0001 according to Student's *t*-test. **H, I)** Flow cytometric analysis and quantification of CD44^+^CD133^+^ cells in AsPC-1/GEM cells treated with peptide CP-SAIDP5 (5 μM, 24 h) or gemcitabine (40 μM, 24 h) alone or in combination. Numbers in the graphs indicate the percentage of this subpopulation (n = 3). ***P < 0.001; ****P < 0.0001 according to Student's *t*-test. Data were expressed as mean ± SD.

**Fig S7. STYK1-driving peptides inhibits gemcitabine-resistant pancreatic cancer development, related with Fig.7. A-D)** Representative images and quantitative analysis of DNA synthesis in PANC-1/GEM and AsPC-1/GEM cells treated with peptide CP-SAIDP5 (5 μM, 24 h) or gemcitabine (5 μM for PANC-1/GEM cells, or 40 μΜ for AsPC-1/GEM cells, 24 h) alone or in combination. Scale bar: 100 μm. **P < 0.01; ***P < 0.001 according to Student's *t*-test. **E, F)** Peptide CP-SAIDP5 (5 μM) or gemcitabine (5 μM for PANC-1/GEM cells, or 40 μΜ for AsPC-1/GEM cells) alone or co-treated PANC-1/GEM and AsPC-1/GEM cells at different times, OD values were determined by CCK8. ****P < 0.0001 according to Student's *t*-test. **G-J)** Representative images and quantitative analysis of wound healing in PANC-1/GEM and AsPC-1/GEM cells treated with peptide CP-SAIDP5 (5 μM) or gemcitabine (5 μM for PANC-1/GEM cells, or 40 μΜ for AsPC-1/GEM cells) alone or in combination. Scale bar: 200 μm. *P < 0.05; **P < 0.01; ***P < 0.001; ****P < 0.0001 according to Student's *t*-test. **K-P)** Concentrations of WBC, RBC, PLT, BUN, AST, ALT in the serum of peptide CP-SAIDP5 or gemcitabine alone or co-treated gemcitabine-resistant PDX mice (n = 4). ns (no significance) according to Student's *t*-test. **Q)** Schematic diagram of the experimental procedure of drug administration by intraperitoneal injection of xenogeneic tumors in nude mice. **R)** Representative images of resected tumors in peptide CP-SAIDP5 (25 mg/kg) or gemcitabine (25 mg/kg) alone or in combination treatment groups (n = 5). **S)** Tumor weights of resected tumors from different groups (n = 5). **P < 0.01; ****P < 0.0001 according to Student's *t*-test. **T)** Tumor growth curves showing the change in volume of resected tumors from different groups (n = 5). **P < 0.01; ***P < 0.001; ****P < 0.0001 according to Student's *t*-test. **U)** Representative H&E staining images and immunohistochemistry images of Ki67, CD44, CD133, LC3, and p62 expressed in resected tumor tissues (n = 5). Scale bar: 50 μm. Data were expressed as mean ± SD, *P < 0.05; **P < 0.01; ***P < 0.001; ****P < 0.0001; ns (no significance) according to Student's *t*-test.

**Fig S8.** Schematic diagram of the mechanism by which AMPK-driven STYK1 phosphorylation promotes gemcitabine resistance via autophagy-dependent cancer stemness in pancreatic cancer.

# **References**

1.	Zhou, C., Liang, Y., Zhou, L., Yan, Y., Liu, N., Zhang, R., Huang, Y., Wang, M., Tang, Y., Ali, D.W., et al., “TSPAN1 promotes autophagy flux and mediates cooperation between WNT-CTNNB1 signaling and autophagy via the MIR454-FAM83A-TSPAN1 axis in pancreatic cancer,” *Autophagy* *17*, (2021): 3175-3195, 10.1080/15548627.2020.1826689.

2.	Zhou, C., Zhu, X., Liu, N., Dong, X., Zhang, X., Huang, H., Tang, Y., Liu, S., Hu, M., Wang, M., et al., “B-lymphoid tyrosine kinase-mediated FAM83A phosphorylation elevates pancreatic tumorigenesis through interacting with β-catenin,” *Signal Transduct Target Ther* *8* (2021): 66, 10.1038/s41392-022-01268-5.

3.	Livak, K.J., and Schmittgen, T.D, “Analysis of relative gene expression data using real-time quantitative PCR and the 2(-Delta Delta C(T)) Method,” *Methods 25*, (2001): 402-408, 10.1006/meth.2001.1262.

**Uncropped/full-size gel/blot for Figure 1**

**Uncropped/full-size gel/blot for Figure S1**

**Uncropped/full-size gel/blot for Figure 2**

**Uncropped/full-size gel/blot for Figure S2**

**Uncropped/full-size gel/blot for Figure 3**

**Uncropped/full-size gel/blot for Figure S3**

**Uncropped/full-size gel/blot for Figure 4**

**Uncropped/full-size gel/blot for Figure S4**

**Uncropped/full-size gel/blot for Figure 5**

**Uncropped/full-size gel/blot for Figure S5**

**Uncropped/full-size gel/blot for Figure 6**

**Uncropped/full-size gel/blot for Figure S6**
